# Eye movement analysis detects early neurological alterations in cirrhotic patients classified as not having minimal hepatic encephalopathy by the Psychometric Hepatic Encephalopathy Score

**DOI:** 10.1371/journal.pone.0358353

**Published:** 2026-09-15

**Authors:** Juan-José Gallego, Franc Casanova-Ferrer, Alessandra Fiorillo, Adrià López-Gramaje, Danna Campos, Amparo Urios, Javier Megías, Teresa San-Miguel, Montserrat Alcahuz-Griñán, Lucía Durbán, Elena Kosenko, Cecilia E. García-Cena, Carmina Montoliu

**Affiliations:** 1 Fundación de Investigación Hospital Clínico Universitario de Valencia-INCLIVA, Valencia, Spain; 2 Departamento de Patología, Facultad de Medicina, Universidad de Valencia, Valencia, Spain; 3 Department of Nursing and Podiateth, Universidad de Valencia, Valencia, Spain; 4 Servicio de Digestivo, Hospital Arnau de Vilanova, Valencia, Spain; 5 Institute of Theoretical and Experimental Biophysics of Russian Academy of Sciences, Pushchino, Russia; 6 Universidad Politécnica de Madrid, Centre for Automation and Robotics, Madrid, Spain; THe University of Texas in Austin, UNITED STATES OF AMERICA

## Abstract

Cirrhotic patients classified by the Psychometric Hepatic Encephalopathy Score (PHES) as without minimal hepatic encephalopathy (MHE) may present subtle cognitive alterations which could be detected by eye movement analysis. The aims were to develop a score for detecting early cognitive alterations in cirrhotic patients using specific psychometric tests and to identify which eye movement parameters could be used to detect them. A total of 118 cirrhotic patients (32 with MHE and 86 without MHE, according to PHES) and 35 healthy controls underwent psychometric assessment and eye movement testing by video-oculography. Forty-six patients without MHE (53.5%) showed impaired cognitive performance and were classified as having early cognitive impairment (early-MHE) using a novel score based on the most affected variables from several psychometric tests. This score was developed using historical cohorts of 519 cirrhotic patients and 74 age-matched healthy controls, and the optimal set of parameters included were from d2, Oral Symbol Digit Modalities, and Stroop tests. Early-MHE patients showed poorer cognitive performance and alterations in eye movement tests, particularly in antisaccade and fixation tasks. These abnormalities were more pronounced in MHE patients, suggesting a continuum of cognitive dysfunction from patients without detectable impairment by PHES to those with MHE. Eye movement variables with the highest predictive value for detecting mild cognitive alterations (early-MHE + MHE) were obtained from horizontal and vertical antisaccade tasks and fixation tests. Multivariate analysis grouping by eye movement task showed that a model including variables from the horizontal antisaccade test achieved the best predictive performance, with AUROC of 0.803 (95% CI: 0.711–0.896; p < 0.0001), 66.7% sensitivity, and 90.6% specificity at a relative-risk cutoff of 65.66%. Eye movement analysis is a rapid, objective, and non-invasive tool for detecting subtle neurological alterations in cirrhotic patients. This approach may facilitate earlier identification of cognitive impairment and support timely clinical intervention.

## Introduction

Minimal hepatic encephalopathy (MHE) is characterized by cognitive and motor impairments, including alterations in cognitive and executive functions such as selective and sustained attention, visuospatial perception and motor speed [1–3]. These impairments negatively affect daily functioning, reduce quality of life and survival, and increase the risk of falls and hospitalizations [4–6]. MHE represents the earliest clinically recognized stage of hepatic encephalopathy (HE) and is considered the main risk factor for the development of overt HE [7–9]. Early identification and treatment of MHE would improve the quality of life, prevent progression to overt HE, reduce healthcare costs, and improve clinical outcomes [10,11].

Since the 11th World Congress of Gastroenterology (1998), the Psychometric Hepatic Encephalopathy Score (PHES) has been widely accepted as the reference standard for the diagnosis of MHE [12,13]. However, over the last decade, several studies have shown that PHES lacks sufficient sensitivity to identify all patients with mild cognitive and motor impairment [14–17]. Butz et al [16] showed that ataxia and some motor abnormalities may represent early markers of cerebral dysfunction in a subgroup of cirrhotic patients before deficits become detectable by conventional psychometric testing. Similarly, several studies have shown that a proportion of patients classified as “without MHE” according to PHES already exhibit impairments in specific neurological functions [3,14–17]. Felipo et al. [3,17] demonstrated that some patients without MHE according to PHES showed deficits in attention assessed by the Stroop test as well as impairments in bimanual coordination. In a more detailed study, Giménez-Garzó et al [14], found that 42% of patients classified as without MHE by PHES exhibited alterations in attention and coordination that were not detected by PHES. In that study, the oral Symbol Digit Modalities Test (SDMT), d2 and bimanual and visuo-motor coordination tests were more sensitive than PHES in detecting these subtle alterations [14]. Moreover, patients classified as without MHE by PHES but presenting these early cognitive alterations showed an increased risk of developing clinical complications, including overt HE [14]. Together, these findings indicate that reliance on PHES alone may leave a substantial proportion of patients with early neurological impairment undetected.

The diagnosis of MHE using psychometric tests also presents practical limitations. These tests are time consuming, require trained personnel, and must be adjusted for age and education level. Consequently, their implementation in routine clinical practice remains limited, and many patients with MHE remain undiagnosed and untreated worldwide. Therefore, there is a need for objective, rapid, reproducible and sensitive tools that facilitate the early detection of cognitive impairment in cirrhotic patients.

Alterations in cognitive processing are often reflected in abnormalities of saccadic eye movements [18]. Accordingly, the analysis of eye movement parameters has been proposed as a valuable approach for the early detection of cognitive and motor dysfunction in several neurological disorders, including multiple sclerosis, Parkinson’s disease, and Alzheimer’s disease [18–21].

Several studies have demonstrated the presence of eye movement abnormalities in patients with cirrhosis and highlighted their potential utility for the diagnosis of MHE and HE [22–24]. These studies reported impairments in smooth pursuit performance and increased saccadic latency, both of which correlated with psychometric performance and the severity of encephalopathy [22–24].

Previously, our group evaluated eye movement alterations in cirrhotic patients with and without MHE using a gaze tracker, based on video-oculography [25,26]. This technique allows to evaluate and analyze with high precision oculomotor responses during a variety of visual tasks [25,26], which are involved in some neurological diseases and their progression. Furthermore, the tasks are simple to perform and well tolerated by patients. In our previous study, we analyzed a large set of eye movement variables and found that patients with MHE exhibited abnormalities in 56 out of 177 measured parameters compared to patients without MHE. These alterations were associated with deficits in attention and mental processing speed [26].

As noted above, the PHES does not detect all cirrhotic patients with early subtle cognitive alterations [14–17]. We therefore performed, in addition to PHES, other psychometric tests assessing different cognitive domains, which are more sensitive to detect these subtle impairments: d2 test (for selective and sustained attention and concentration), oral SDMT (mental processing speed) and Stroop test (cognitive flexibility, selective attention, inhibition) [3,14]. It would be useful to have a score that allows for the quantification of these alterations and the classification of patients for the subsequent evaluation of eye movement parameters.

The first aim of this study was to develop a score that allowed us to identify and stratify cirrhotic patients with early cognitive alterations who are not classified as having MHE by PHES. We refer to this subgroup as early-MHE. A second aim was to perform a comprehensive analysis of 164 eye movements parameters obtained from a set of visual tests (visually-guided saccades, memory-guided saccades, antisaccades, smooth pursuit eye movements and fixation test) recorded by video-oculography, in order to identify a set of parameters capable of detect early neurological alterations in cirrhotic patients that are not detectable using PHES.

## Patients and methods

### Subjects

This cross-sectional study included 118 patients with liver cirrhosis, who were consecutively recruited between 26 April 2018–12 July 2024 at the outpatient clinics at the Hospital Clínico and Hospital Arnau de Vilanova (Valencia, Spain). Inclusion criteria were patients older than 18 years and a diagnosis of liver cirrhosis of any etiology established by liver histology or a combination of clinical, biochemical, and imaging features. Exclusion criteria were current or previous overt HE, alcohol consumption within the previous 6 months, infection, recent antibiotic use (over 6 weeks), gastrointestinal bleeding, use of drugs that affect cognitive function, hepatocellular carcinoma and the presence of neurological or psychiatric disorders. Patients included in the study did not show fever or any clinical or biological sign of recent infection. Thirty-five healthy volunteers were also recruited as a control group. Patients and controls underwent clinical evaluation, psychometric assessment, and blood analyses to determine ammonia levels and routine biochemical parameters on the same day. The study protocols were approved by the Scientific and Ethical Committees of Hospital Clínico Universitario and Arnau de Vilanova Hospital of Valencia, Spain (approval code: 2018.051; approval date: 27 February 2018; approval code: 2023/130; approval date: 29 February 2024) and were in accordance with the principles of the World Medical Association’s Declaration of Helsinki, the Council of Europe Convention regarding human rights and the requirements established in Spanish legislation in the field of Biomedical research, personal data protection and bioethics. Written informed consent was obtained from all participants prior to enrollment.

### Diagnosis of MHE and psychometric assessment

Thirty-two patients were diagnosed with MHE using the Psychometric Hepatic Encephalopathy Score battery (PHES) [12]. PHES scores were adjusted for age and education level using Spanish normative data (www.redeh.org/TEST_phes.htm). Patients with a PHES score ≤ −4 were classified as having MHE. Healthy volunteers also completed the PHES battery to exclude cognitive impairment according to this criterion. To assess specific cognitive and motor domains, participants additionally underwent the Stroop test (selective attention, cognitive flexibility and inhibitory mental control), the oral Symbol Digit Modalities test (Oral SDMT; mental processing speed and selective attention); d2 test (selective and sustained attention and concentration), Digit Span and letter-number sequencing tests (working memory), and bimanual and visuomotor coordination tests. All psychometric tests were administered and scored as previously described in Giménez-Garzó et al [14].

### Analysis of eye movements

Eye movement analysis was performed using a gaze tracker system (OSCANN desk100, AURA Innovative Robotics) based on the video-oculography, and analyzed with OSCANN software [25,26]. Eye movement assessment was performed after psychometric testing. Ten eye movement tasks were evaluated in the following order: visually-guided saccades, memory-guided saccades, antisaccades, and smooth pursuit, each in horizontal and vertical versions; sinusoidal smooth pursuit and fixation tests were also included.

A detailed description of the procedure has been reported previously [26]. Briefly, during the visually guided saccade task, a target was presented at the center of the screen for 1500 ms and then moved to a randomly selected position on either side of the screen before returning to the center. Participants were instructed to follow the target as quickly and accurately as possible. Each horizontal and vertical task lasted 36 s.

In the memory guided saccade task, participants were required to remember the position of the stimulus. After presentation of the peripheral target, it disappeared for 1500 ms, and participants were instructed to direct their gaze to the remembered target position. Each task lasted 72 s.

During the anti-saccade task, the target followed the same movement pattern as in the visually guided saccade task. However, participants were instructed to direct their gaze towards the position of the screen opposite of the stimulus. Each task lasted 36 s.

In the smooth pursuit task, the target moved continuously across the screen with a period of 8 s, and participants were instructed to follow the stimulus all time. The task duration was 32 s. An additional horizontal smooth pursuit condition using sinusoidal velocity changes was also included.

In the fixation task, participants were instructed to maintain their gaze on the immobile stimulus, presented at the center of the screen for 20 s.

The eye movement variables obtained from each task are summarized in Table 1. For several eye-movement parameters, a corresponding variability measure (standard deviation) was also calculated (e.g., latency and SD of latency).

**Table 1:** Main variables measured in eye-movement tests performed.

Visual saccades and Memory-guided saccades (horizontal and vertical versions)	
Latency (ms)	Time elapsed between the change of the stimulus and the first ocular movement performed by the volunteer in response to the stimulus change
Return latency (ms)	Elapsed time to initiate the movement to return to the center
Number of correct saccades	Number of correct visual saccades during the test
Gaze Error (º):	Difference between the establishment of the gaze and the final position of the stimulus. If Error > 0 the saccade is hypermetria and if Error < 0 is hypometria.
**Antisaccades (horizontal and vertical versions)**	
Latency (ms)	Time between the stimulus change and the first anti-saccadic movement, during the correct visual anti-saccade
Latency of reflexive saccades (ms)	Time between the stimulus change and the first anti-saccadic eye movement that crosses the center of the monitor during a reflexive visual anti-saccade.
Duration of reflexive saccades (ms)	Fixation time during the reflexive saccade, that is, time between the first point of fixation of the reflexive saccade and the first point of fixation of the anti-saccade that crosses the center of the monitor
Gaze Error (º)	Difference between the establishment of the gaze and the final position of the stimulus. If Error > 0 the saccade is hypermetria and if Error < 0 is hypometria.
Number of correct antisaccades	Number of times that an anti-saccade is made without the presence of a pro-saccade during a visual anti-saccade during the test
Number of incorrect antisaccades	Number of incorrect saccades
Number of anticipated antisaccades	Number of early visual saccades during the test
Return latency (ms)	Elapsed time to initiate the movement to return to the center
Return peak of velocity	Maximum speed of the gaze in the saccadic movement of returning to the center
**Smooth pursuit (horizontal, vertical and sinusoidal versions)**	
Cath-up saccades	Number of saccadic movements made in the sense of stimulus.
Back-up saccades	Number of saccadic movements made in the opposite sense of stimulus
Square wave jerks	Square Wave Jerks are defined by two opposite saccadic movements whose amplitude are between 0.2º to 5º
Pursuit time (%)	Percentage in which smooth pursuit is performed.
Total mean squared error of position	Square Mean Error is the difference between the position of the stimulus and the gaze.
Pursuit mean squared error of position	
Gain	Measure of the speed matching, being the gain >1 when the eye anticipates to the stimulus movement, and the gain <1 when the eye is delayed.
**Fixation**	
Blinks	Number of blinks
Saccades	Number of saccades performed during the test.
Biphasic- square wave jerks	Number of Square Wave Jerks
BCEA (º)	The Bivariate Contour Ellipse Area (BCEA) is defined by the 69% of the fixation points. This variable represents the precision of the fixation during the test.
Ox (horizontal SD) (º)	Horizontal standard deviation of fixation points of the gaze
Oy (vertical SD) (º)	Vertical standard deviation of fixation points of the gaze
Centroid x (horizontal) (º)	Symmetry Center of BCEA in horizontal
Centroid y (vertical) (º)	Symmetry Center of BCEA in vertical
Microsaccades amplitude (º)	Microsaccade is an eye movement characterized by amplitude value of between 0.03 and 1 degrees and a duration value of 10–30 milliseconds
Velocity of microsaccades (º/s)	Mean of velocity of microsaccades performed during the test
Peak velocity-microsaccades (º/s)	Mean of peak velocity of microsaccades performed during the test.
Drift amplitude (º)	A drift is a slow movement that occurs when the gaze is fixed in a stimulus and it is placed between two microsaccades. Drift amplitud corresponds to mean of the amplitudes of drift performed during the test

SD, standard deviation ms, milliseconds, s, seconds

### Development of a methodology for identifying early cognitive impairment in patients with liver cirrhosis

Results from a historical cohort of 74 age-matched healthy controls were used to establish reference values for cognitive performance in the psychometric tests employed in this study. Cognitive impairment was defined using the mean ± 2 standard deviations (SD) of the control population as the normality criterion. This criterion was subsequently applied to a historical cohort of 519 patients with cirrhosis. The proportion of patients showing impairment in each psychometric variable was calculated, and the tests with the highest frequency of abnormalities were selected. Based on these variables, and following the methodology described by Adam and Foley [27], a composite score was developed to identify early cognitive impairment in patients with liver cirrhosis.

### Statistical analyses

Values are given as mean ± standard error of the mean (SEM) for parametric variables; in case of nonparametric variables, values are given as median and interquartile range (IQR) unless otherwise stated. Results were analyzed using one of three options: one-way ANOVA followed by post-hoc Tukey’s multiple comparison test for variables both parametric and homoscedastic, Welch’s ANOVA followed by Games-Howell’s multiple comparison test for variables parametric but not homoscedastic, and Kruskal–Wallis’ test followed by Dunn’s test for nonparametric variables. Due to the number of variables analyzed in this study, p values obtained were corrected using the false discovery rate (FDR) method [28]. FDR values < 0.05 were considered significant. For all statistical analyses and receiver operating characteristic (ROC) curves, data were processed and analyzed using the software R version 4.1.1. Finally, to test the discriminant capabilities of the parameters from the eye-movements tests that showed the best individual results during the analysis of their ROC curves, several multivariate generalized linear models were created using the *glm* function included in the stats package (version 3.6.2) for R. Internal validation using bootstrap resampling with 1,000 iterations and optimism correction was performed as recommend the TRIPOD statement [29].

## Results

### Classification of early-MHE patients

Previous studies have shown that a substantial proportion of cirrhotic patients classified as not having MHE according to PHES exhibit subtle cognitive alterations that are not detected by this test battery [14]. To identify these patients, referred to here as early-MHE, we developed a psychometric score based on the cognitive tests included in the present study.

Reference values were established using a cohort of healthy controls (n = 74) by applying the mean ± 2 standard deviations (SD) criterion to each psychometric variable. The resulting cutoff values are shown in Table 2. Participants with values outside these limits were considered to have impairment in the cognitive or motor function assessed by the corresponding parameter.

**Table 2:** Proportion of cirrhotic patients showing cognitive impairment for each parameter, and formulas of early-MHE score.

Psychometric test	Control mean	SD	Cutoff impairment (Control mean ± 2SD)	Percentage of patients with impairment
**Stroop Test**				
Congruent task	50.82	8.59	33.65	16.55
Neutral task	50.55	8.85	32.85	15.46
Incongruent task	50.09	8.91	32.28	**18.22**
**d2 Test**				
TR	58.66	16.42	25.81	33.33
TA	61.73	16.35	29.04	31.17
O	54.15	27.33	108.81	0.00
C	61.19	23.73	108.66	0.00
TOT	60.92	16.42	28.07	**34.88**
CON	64.23	14.97	34.29	**40.74**
**Oral SDMT**				
Correct answer	12.74	2.00	8.73	**29.14**
**Digit span test**				
Forward task	9.38	2.11	5.17	11.53
Backward task	6.06	2.04	1.97	0.94
**Letter-number sequencing test**				
Correct series	9.76	1.81	6.15	17.97
**Psychometric test: parameter**			**Formulas of early-MHE score**	
d2 test: CON			Ratio of (patient result – 64.23)/14.97)	
d2 test: TOT			Ratio of (patient result – 60.92)/16.42)	
Oral SDMT: Correct answer			Ratio of (patient result – 12.74)/2.00)	
Stroop test: Incongruent task			Ratio of (patient result – 50.09)/8.91)	
*Example*				
**Psychometric test: parameter**		**Raw result**	**Formulas**	**Score**
d2 test: CON		20.00	Ratio of (patient result – 64.23)/14.97)	-2.00
d2 test: TOT		15.00	Ratio of (patient result – 60.92)/16.42)	-2.00
Oral SDMT: Correct answer		10.00	Ratio of (patient result – 12.74)/2.00)	-1.00
Stroop test: Incongruent task		36.00	Ratio of (patient result – 50.09)/8.91)	-1.00
**Final early-MHE score**				**-6.00**

SD, standard deviation; TR, total responses; TA, total right answer; O, omission errors; C, commission errors; TOT, effectiveness index; CON, concentration index; SDMT, symbol digit modalities test; MHE, minimal hepatic encephalopathy.

These cutoffs were subsequently applied to a historical cohort of 519 patients with cirrhosis to determine the proportion of patients showing impairment in each psychometric variable. To construct the early-MHE score, we selected variables according to two criteria: (i) a high prevalence of impairment among cirrhotic patients and (ii) assessment of distinct cognitive domains. The variables showing the highest frequency of impairment were the concentration index (CON) of d2 test (40.7%), the total performance score (TOT) of the d2 test (34.9%), the number of correct responses in the Oral SDMT (29.1%), and incongruent task score of Stroop test (18.2%) (Table 2). These variables assess complementary cognitive functions, including concentration, selective and sustained attention, and mental processing speed.

Using the Adam and Foley method [27], individual scores were calculated for each patient and each psychometric parameter, as the number of standard deviations by which the patient’s value deviated from the mean of healthy controls, according to the formulas shown in Table 2. These standardized values were summed to obtain a composite score for each participant. Patients without MHE were classified as having early-MHE when the composite score was ≤ −4. A score of −4 therefore represents an overall cognitive performance approximately one standard deviation below the mean across the selected tests. This additive approach ensures that classification is based on consistent impairment across domains rather than isolated abnormalities in a single test. Because of its structure, different patterns of impairment may result in the same final score, including mild deficits across several domains or more pronounced impairment in specific tests with preserved performance in others. This approach allows for the identification of heterogeneous patterns of early cognitive dysfunction while maintaining consistency with previously established composite scoring systems for MHE, such as PHES [12] and cognitive impairment scores in patients with MASLD (Metabolic Dysfunction-Associated Steatotic Liver Disease) [30].

Using this criterion, 46 of 86 (53.5%) patients without MHE were classified as having early cognitive impairment (early-MHE).

### Demographic, clinical, and psychometric characteristics of patients and controls

Table 3 shows demographic, clinical, and psychometric characteristics of control and patient groups. Thirty-two patients were classified as having MHE according to the PHES battery, and 46 of 86 (53.5%) patients without MHE were classified as early-MHE patients, according to the new score. No significant differences were observed between groups in age, etiology of cirrhosis, or MELD score. A higher proportion of Child–Pugh B patients was observed in the MHE compared with the NMHE group (p < 0.05), although most patients in all groups were classified as Child-Pugh A. There is also a difference in the proportion of sexes in the studied population, due to the higher incidence of liver cirrhosis in males (Table 3). Regarding the neuropsychological assessment, both early-MHE and MHE patients showed significantly worse performance than controls and NMHE patients in the early-MHE composite score and in all individual tests, except in the Letter-number sequencing test, in which early-MHE patients differed only from controls (Table 3). In addition, MHE patients performed significantly worse than early-MHE patients in PHES, the incongruent task of the Stroop test, the oral SDMT, and the forward Digit span test (Table 3).

**Table 3:** Demographic and clinical characteristics and psychometric performance of controls and patients.

Variable	Control (n = 35)	NMHE (n = 40)	Early-MHE (n = 46)	MHE (n = 32)	Global FDR values
**Demographic characteristics**					
Sex (M/F)	13/22	32/8	41/5	24/8	p < 0.001
Age (years)	61.5 ± 1.7	62.2 ± 1.4	62.6 ± 1.3	64.5 ± 1.5	0.559
**Clinical characteristics**					
**Etiology of cirrhosis (n)**					
Alcohol		14	20	14	0.473
HBV/HCV		7	12	5	
MASLD		11	7	9	
Others		8	7	4	
Child-Pugh score (A/B/C)		37/3/0	36/10/0	22/10/0 ^α^	0.036
MELD score		8.2 ± 0.4	9.4 ± 0.5	9.7 ± 0.5	0.118
**DM** (Yes/No)		4/36	13/33	8/24	0.097
**Psychometric performance**					
**PHES**	0.75 ± 0.28	0.18 ± 0.23	−1.09 ± 0.19^***/αα^	−7.1 ± 0.57^***/ααα/βββ^	p < 0.001
**Early-MHE score**	1.42 ± 0.59	−0.61 ± 0.46	−7.37 ± 0.38^***/ααα^	−10.3 ± 0.50^***/ααα^	p < 0.001
**Stroop test**					
Congruent task	50.3 ± 1.6	49.8 ± 1.4	42.7 ± 1.2^**/αα^	34.8 ± 1.4^***/ααα^	p < 0.001
Neutral task	51.0 ± 1.8	50.3 ± 1.3^***^	41.9 ± 1.1^***/ααα^	33.5 ± 1.2^***/ααα^	p < 0.001
Incongruent task	50.8 ± 2.2	52.2 ± 1.5^***^	40.5 ± 1.4^**/ααα^	34.5 ± 1.3^***/ααα/ββ^	p < 0.001
**d2 test**					
TR	60.1 ± 4.1	52.9 ± 2.8	30.0 ± 2.2^***/ααα^	25.2 ± 2.9^***/ααα^	p < 0.001
TA	65.6 ± 4.0	58.7 ± 2.7	30.9 ± 1.9^***/ααα^	23.2 ± 3.1^***/ααα^	p < 0.001
O	55.1 ± 5.0	61.5 ± 4.6	49.2 ± 4.4	46.7 ± 6.0	0.196
C	60.2 ± 4.3	58.7 ± 4.1	39.5 ± 4.3^***/αα^	29.8 ± 5.6^***/ααα^	p < 0.001
TOT	59.9 ± 4.9	56.6 ± 2.8	28.0 ± 2.0^***/ααα^	22.6 ± 2.9^***/ααα^	p < 0.001
CON	67.7 ± 3.6	58.0 ± 3.0	30.3 ± 2.3^***/ααα^	22.1 ± 3.7^***/ααα^	p < 0.001
**Oral SDMT**					
Scaled score	13 ± 0.55	12.19 ± 0.36	9.54 ± 0.32^***/ααα^	7.47 ± 0.44^***/ααα/ββ^	p < 0.001
**Digit span test**					
Forward task	9.17 ± 0.51	8.86 ± 0.44	7.6 ± 0.27^*/α^	6.04 ± 0.28^***/ααα/ββ^	p < 0.001
Backward task	5.96 ± 0.59	5.46 ± 0.3	4.12 ± 0.25^**/αα^	4.07 ± 0.3^**/αα^	0.001
**Letter-number sequencing test**					
Correct series	9.71 ± 0.46	7.77 ± 0.5	6.08 ± 0.46^***^	4.93 ± 0.61^***/αα^	p < 0.001

Values are the mean ± SEM. Values significantly different from the control group are indicated by asterisks (*), from NMHE patients by α, and from early-MHE by β (^*/α^ p < 0.05; ^**/αα/ββ^ p < 0.01; ^***/ααα/βββ^ p < 0.001). NMHE, MHE, patients without or with minimal hepatic encephalopathy respectively; early-MHE, NMHE patients with cognitive alterations according to test other than PHES (see material and methods section); M, male; F, female, HBV, hepatitis B virus; HCV, hepatitis C virus; MASLD, metabolic dysfunction–associated steatotic liver disease; PHES, psychometric hepatic encephalopathy score; MELD, Model for End-stage Liver Disease; DM, Diabetes mellitus; TR, total responses; TA, total right answer; O, omission errors; C, commission errors; TOT, effectiveness index; CON, concentration index; SDMT, symbol digit modalities test. d2 parameters are expressed as scaled score. Digit span results are expressed as correct series performed.

### Performance in eye movement tests

A total of 164 eye movement variables were extracted from the 10 experimental tasks (S1-S5 Tables). The variables analyzed are summarized in Table 1, and those showing statistically significant differences are presented in Tables 4-6.

**Table 4:** Results of eye movement parameters in visual saccades, memory-guided saccades and smooth pursuit tests.

Variable	Control	NMHE	Early-MHE	MHE	Global FDR values
**Horizontal visual saccades**					
Latency (ms)	225.0 (26.3)	222.1 (34.1)	232.5 (36.6)	247.4 (60.7)^*^	ns
SD of latency	37.6 (20.4)	35.6 (29.5)	34.4 (17.5)	48.6 (51.0)^*/α/β^	ns
Return latency (ms)	201.0 (33.6)	212.3 (23.8)	209.0 (33.4)	239.0 (47.4)^*/β^	ns
**Vertical visual saccades**					
Latency (ms)	246.6 (33.0)	245.6 (45.6)	258.9 (36.2)^*^	275.6 (55.6)^*/α^	ns
Return latency (ms)	221.4 (39.4)	226.3 (39.5)	243.2 (45.2)^*^	261.1 (52.4)^***/α^	0.010
SD of return latency	29.6 (18.9)	34.0 (27.9)^*^	41.3 (30.3)^**^	42.1 (39.6)^*^	ns
**Horizontal memory-guided saccades**					
Latency (ms)	288.1 (87.9)	339.9 (136.5)^*^	318.2 (118.9)^*^	372.0 (168.4)^**^	ns
SD of latency	82.7 (85.0)	112.9 (114.8)	127.2 (123.3)^*^	127.4 (91.7)	ns
Gain	1.05 (0.13)	1.00 (0.12)	1.02 (0.19)	0.97 (0.19)^*^	ns
Number of correct saccades	11 (2)	11 (4)	10 (7)^*/α^	4 (6)^***/ααα/β/§^	p < 0.001
**Vertical memory-guided saccades**					
Latency (ms)	244.4 (81.2)	324.2 (155.9)^**^	296.6 (140.6)^*^	422.0 (248.0)^***/α/β^	0.003
SD of latency	51.45 (56.4)	101.1 (144.4)	94.9 (112.0)^*^	149.8 (129.6)	ns
Positive error	1.51 (0.98)	1.06 (1.11)	0.91 (0.71)	2.13 (1.02)^β^	ns
Number of correct saccades	8 (1)	8 (2)	6 (6)^**^	2 (3)^***/ααα/ββ^	p < 0.001
Return latency (ms)	241.0 (90.9)	271.2 (106.2)	286.4 (158.8)^**^	317.5 (143.2)^*^	ns
**Horizontal smooth pursuit**					
Back-up saccades	0 (0)	0 (1)	0 (1)	1 (2)^**/α/β^	0.048
Pursuit time (%)	92.5 (5.5)	92.1 (5.5)	91.1 (6.1)^§^	88.3 (4.5)^*/α/§^	ns
Total mean squared error of position	1.98 (1.51)	2.32 (1.54)	2.59 (2.68)^*/§^	3.19 (3.56)^*/α/§^	0.048
Pursuit mean squared error of position	1.96 (1.41)	2.25 (1.56)	2.53 (2.61)^*/§^	2.99 (3.47)^*/α/§^	0.048
Gain	0.87 (0.17)	0.89 (0.29)	0.79 (0.19)^*^	0.73 (0.14)^**/αα/§^	0.024
**Vertical smooth pursuit**					
Cath-up saccades	13 (12)	14 (7)	15 (4)^§^	18 (6)^*/α/§^	0.041
Square wave jerks	1 (2.5)	2 (4)	2 (5)^*^	3 (3)^*^	0.041
Pursuit time (%)	93.1 (5.6)	92.2 (4.9)	91.8 (6.9)^§^	90.0 (9.0)^*/α/§^	ns
Total mean squared error of position	1.50 (1.64)	1.61 (1.98)	1.86 (2.45)	2.72 (2.02)^**/αα^	0.016
Pursuit mean squared error of position	1.44 (1.49)	1.50 (1.66)	1.78 (1.97)	2.63 (1.9)^**/αα^	0.016
Gain	0.83 (0.23)	0.83 (0.20)	0.79 (0.20)^§^	0.70 (0.18)^**/α/§^	0.033
**Sinusoidal smooth pursuit**					
Cath-up saccades	19 (14)	16 (11)	16 (8)^§^	21 (6)^α/§^	ns
Latency (ms)	259.7 (36.0)	264.2 (51.6)	272.0 (52.7)	308.3 (77.7)^*^	ns
Total mean squared error of position	1.65 (2.80)	2.24 (2.21)	2.52 (2.02)	3.86 (2.94)^*/α^	ns
Pursuit mean squared error of position	1.63 (2.69)	2.11 (2.12)	2.35 (1.82)	3.72 (2.83)^*/α^	ns
Gain	0.86 (0.22)	0.83 (0.36)	0.76 (0.33)	0.67 (0.30)^α^	ns

Values are expressed as median (interquartile range). Data were analyzed using Kruskal–Wallis’ test followed by Dunn’s test for comparisons between groups, and p values obtained were corrected using the false discovery rate (FDR) method. Values significantly different from the control group are indicated by asterisks (*), from NMHE patients by α, and from early-MHE by β (^*/α/β^ p < 0.05; ^**/αα/ββ^ p < 0.01; ^***/ααα^ p < 0.001). ^§^ Significant interaction between severity of liver damage (Child Pugh and/or MELD scores) and variable tested (see S11-S13 Tables). NMHE, MHE, patients without or with minimal hepatic encephalopathy, respectively; early-MHE, NMHE patients with cognitive alterations according to test other than PHES (see Patients and methods section); SD, standard deviation.

**Table 6:** Diagnostic accuracy of eye movement variables to detect mild cognitive alterations in cirrhotic patients by ROC analyses.

Variable	Test	AUROC (95% CI)	p value	Cutoff	Sens.	Spec.
Latency of reflexive saccades	HA	0.772 (0.678 - 0.866)	p < 0.001	676.75^a^	54.84	88.24
Drift amplitude	F	0.747 (0.602 - 0.892)	0.002	0.16^b^	88.10	57.89
Number of incorrect antisaccades	HA	0.734 (0.641 - 0.826)	p < 0.001	1.00	67.14	77.78
Number of anticipated antisaccades	HA	0.734 (0.635 - 0.833)	p < 0.001	1.00	65.71	69.44
SD of negative error	HA	0.734 (0.613 - 0.855)	0.001	2.55	75.00	80.00
Duration of reflexive saccades	HA	0.730 (0.628 - 0.832)	p < 0.001	276.09^a^	63.93	73.53
BCEA	F	0.722 (0.617 - 0.828)	p < 0.001	0.68^b^	66.18	72.73
Latency of reflexive saccades	VA	0.721 (0.613 - 0.829)	0.001	818.86^a^	35.19	100.00
SD of latency of reflexive saccades	HA	0.720 (0.610 - 0.830)	0.001	165.89	66.67	71.88
Ox (horizontal standard deviation)	F	0.718 (0.609 - 0.827)	p < 0.001	0.24^b^	79.41	57.58
SD of duration of reflexive saccades	HA	0.710 (0.594 - 0.826)	0.001	123.03	72.55	68.75
Positive error	VA	0.704 (0.583 - 0.826)	0.004	3.11	42.86	89.66

Receiver operating characteristic (ROC) curves for sensitivity (Sens.) and specificity (Spec.) to detect early cognitive alterations in the cohort of cirrhotic patients using eye movement parameters. Table shows variables with AUROC > 0.7. ^a^: ms; ^b^: º. AUROC, area under the receiver operating curve; CI, confidence interval; Sens: sensitivity; Spec: specificity; BCEA, bivariate contour ellipse area; SD, standard deviation; HA, horizontal antisaccades; F, fixation; VA, vertical antisaccades.

In the visually guided saccade and memory-guided saccade tasks, latency-related parameters were more markedly affected in patients with MHE compared with the other study groups (Table 4). Patients with early-MHE also showed increased latency compared with controls in both tasks. In addition, they exhibited a lower number of correct saccades than both controls and patients without MHE (NMHE) in the vertical memory-guided saccade task. In this same task, patients with MHE showed significantly reduced performance compared with all other groups.

In smooth pursuit tasks, patients with MHE showed impairments compared to controls and NMHE patients in several parameters, including the percentage of pursuit time in both horizontal and vertical conditions, as well as the total and pursuit mean squared error of position, and gain, across the three task variants (Table 4). Patients classified as early-MHE presented impairments compared to controls, with increased total and mean squared error of position and reduced gain in the horizontal smooth pursuit task, as well as increased square wave jerks in the vertical smooth pursuit task. Catch-up saccades were significantly increased in MHE patients compared to NMHE group in both vertical and sinusoidal smooth pursuit tasks (Table 4).

The antisaccade tasks, both horizontal and vertical, showed the highest number of abnormal parameters in patients with MHE and early-MHE (Table 5).

**Table 5:** Results of eye movement parameters in horizontal and vertical antisaccades, and fixation tests.

Variable	Control	NMHE	Early-MHE	MHE	Global FDR values
**Horizontal antisaccades**					
SD of latency	82.4 (69.7)	61.6 (66.8)	173.1 (153.3)^*/α^	81.3 (101.6)	0.050
Latency of reflexive saccades (ms)	550.5 (162.7)	551.0 (205.7)	673.1 (331.4)^**/ααα^	727.8 (359.8)^***/ααα^	<0.001
SD of latency of reflexive saccades	120.4 (68.9)	124.5 (100.0)	180.5 (191.3)^*/αα^	212.3 (91.7)^**/αα^	0.005
Duration of reflexive saccades (ms)	194.4 (90.0)	215.2 (186.3)	306.3 (273.0)^**/αα^	345.4 (285.8)^***/ααα^	<0.001
SD of duration of reflexive saccades	112.6 (72.8)	99.5 (103.4)	183.0 (150.1)^*/αα^	194.6 (90.7)^**/αα^	0.005
Positive error	2.09 (2.58)	2.08 (2.22)	3.54 (5.22)^*^	5.28 (4.53)^**/αα^	0.007
SD of positive error	0.909 (1.180)	1.680 (2.612)	2.366 (2.588)^*^	3.60 (3.07)^***/α^	0.003
Negative error	−2.11 (2.30)	−2.49 (2.12)	−4.03 (7.27)^*^	−4.65 (6.40)^**/α^	0.018
SD of negative error	1.62 (2.10)	1.49 (1.03)	3.14 (4.34)^*/α^	4.97 (6.96)^**/αα^	0.003
Number of correct antisaccades	3 (6)	3 (5)	1 (3)^**/αα^	0 (2)^**/ααα^	p < 0.001
Number of incorrect antisaccades	0 (1)	0 (0)	1 (3)^**/αα^	3 (6)^**/ααα^	p < 0.001
Number of anticipated antisaccades	0 (1)	0 (1)	1 (3)^*/αα^	1 (3)^*/α^	0.008
Return latency (ms)	320.4 (147.3)	362.3 (182.6)	399.4 (154.9)^*^	358.7 (168.4)	ns
SD of return latency	99.4 (55.6)	86.7 (89.6)	152.0 (126.8)^*/α^	157.7 (148.1)	0.022
**Vertical antisaccades**					
Latency (ms)	393.1 (67.9)	397.6 (140.8)	430.0 (167.3)	583.9 (242.1)^**/αα/β^	0.012
Latency of reflexive saccades (ms)	527.4 (104.9)	523.6 (201.0)	580 (160.3)	959.2 (521.0)^***/ααα/ββ^	0.001
Duration of reflexive saccades (ms)	173.6 (63.4)	200.8 (108.8)	222.2 (125.3)	271.9 (359.8)^*/α^	ns
Positive error	1.59 (1.43)	1.54 (1.82)	2.09 (2.99)^*/α^	3.11 (1.96)^**/αα/§^	0.010
SD of positive error	0.88 (0.97)	1.18 (1.46)	1.04 (1.11)	2.09 (1.21)^*/§^	ns
Negative error	−2.51 (2.40)	−2.35 (2.75)	−3.67 (3.87)	−5.47 (6.05)^**/αα/β^	0.026
SD of negative error	1.45 (1.77)	1.40 (2.04)	1.26 (2.01)	4.34 (5.04)^*/α/β^	ns
Number of correct antisaccades	2 (4)	2 (2)	1 (2)^*/α/§^	1 (2)^*/α/§^	0.016
Number of corrected antisaccades	3 (4)	4 (4)	3 (4)	2 (3)^*/α^	ns
Number of incorrect antisaccades	0 (1)	0 (1)	1 (2)^*/α/§^	1 (3)^***/ααα/β^	0.002
Number of anticipated antisaccades	0 (1)	0 (1)	0 (2)	1 (2)^**/α^	0.028
SD of return peak of velocity	112.9 (60.2)	93.4 (51.7)	98.2 (60.1)	145.1 (57.3)^α^	ns
**Fixation**					
Blinks	3 (4)	2 (5)	2 (4)	5 (5)α	ns
Saccades	2 (3)	1 (4)	3 (5)^*/α/§^	4 (6)^*/α^	ns
Biphasic- square wave jerks	0 (0)	0 (1)	0 (2)	1 (3)^*/α^	ns
BCEA (º)	0.44 (0.46)	0.31 (0.69)	1.04 (2.28)^*/αα^	1.39 (2.50)^**/ααα/β^	0.003
Ox (horizontal standard deviation) (º)	0.27 (0.12)	0.23 (0.15)	0.39 (0.81)^**/αα^	0.47 (0.50)^**/αα^	0.006
Oy (vertical standard deviation) (º)	0.22 (0.19)	0.22 (0.32)	0.35 (0.70)	0.43 (0.37)^*/α^	ns
Centroid x (horizontal) (º)	0.08 (0.26)	0.26 (0.43)	0.24 (0.46)^*^	0.12 (0.20)^*/α/β^	ns
Centroid y (vertical) (º)	0.26 (0.33)	0.40 (0.46)	0.54 (0.62)	0.72 (0.78)^*^	ns
Microsaccades amplitude (º)	0.34 (0.16)	0.36 (0.22)	0.42 (0.18)^*^	0.44 (0.10)^**/α^	0.046
Velocity of microsaccades (º/s)	13.8 (6.1)	14.4 (6.6)	14.5 (3.16)	16.3 (2.67)^*/α^	ns
Peak velocity-microsaccades (º/s)	24.5 (10.9)	24.5 (13.5)	26.8 (8.4)	^2^8.8 (5.9)^*/α^	ns
Drift amplitude (º)	0.19 (0.21)	0.14 (0.13)	0.24 (0.14)	0.29 (0.11)^αα^	ns

Values are expressed as median (interquartile range). Data were analyzed using Kruskal–Wallis’ test followed by Dunn’s test for comparisons between groups, and p values obtained were corrected using the false discovery rate (FDR) method. Values significantly different from the control are indicated by an asterisk (*), from NMHE patients by α, and from early-MHE by β ^(*/α/β^ p < 0.05; ^**/αα/ββ^ p < 0.01; ^***/ααα^ p < 0.001). ^§^ Significant interaction between severity of liver damage (Child Pugh and/or MELD scores) and variable tested (see S14 and S15 Tables). NMHE, MHE, patients without or with minimal hepatic encephalopathy, respectively; early-MHE, NMHE patients with cognitive alterations according to test other than PHES (see Patients and methods section); SD, standard deviation; BCEA, bivariate contour ellipse area.

**Fig 1:**
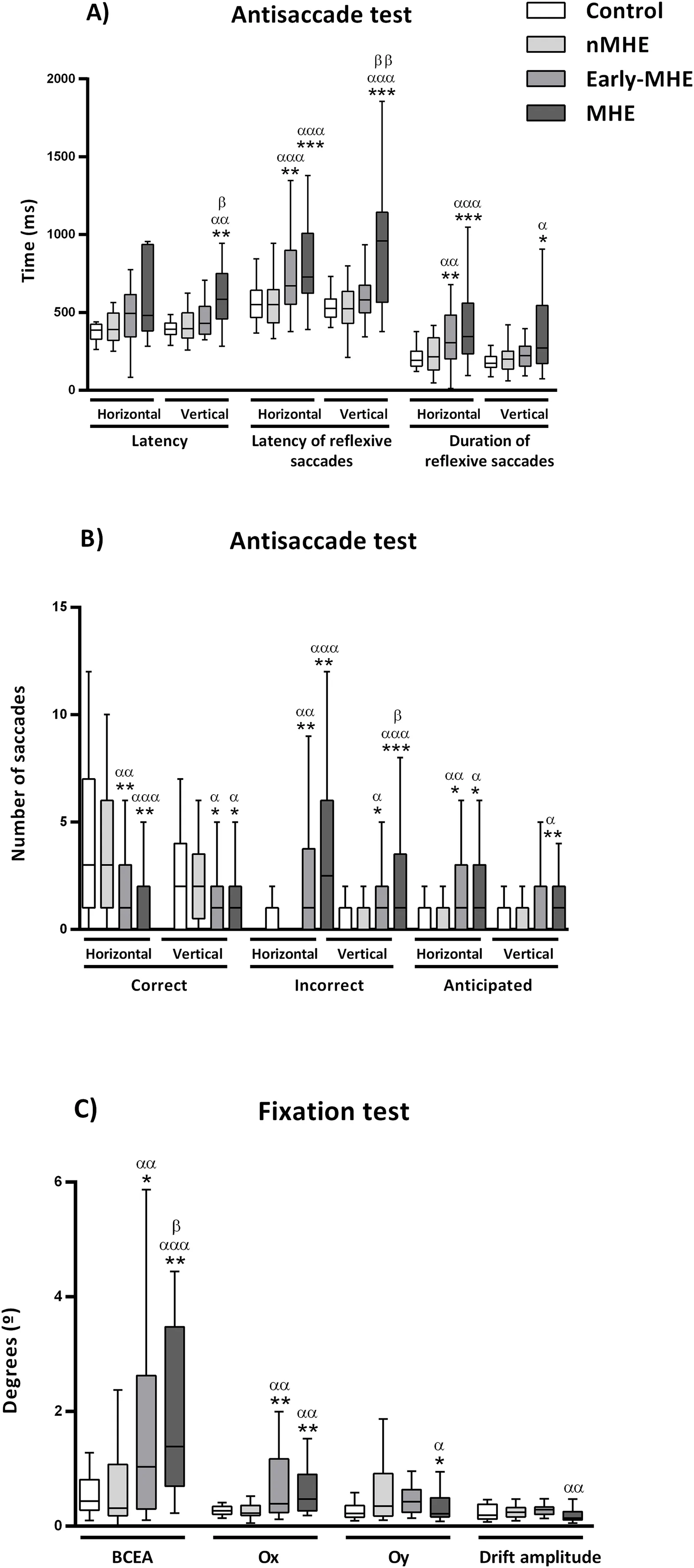
Eye movement test performance in control and cirrhotic patients. A) latency of antisaccades and latency and duration of reflexive saccades in antisaccades test. B) Number of correct, incorrect and anticipated saccades in antisaccades test. C) BCEA, Ox, Oy, and drift amplitude in fixation test.

In the horizontal antisaccade task, most parameters were significantly altered in both early-MHE and MHE patients compared with controls and NMHE patients. These included latency and duration of reflexive saccades and their standard deviations (SD) (Fig 1A; Table 5). The number of correct and incorrect antisaccades were also significantly affected in early-MHE and MHE patients in both horizontal and vertical versions of antisaccade task, whereas anticipated antisaccades were only altered in horizontal version in both groups (Fig 1B; Table 5).

In the vertical antisaccade task, MHE patients showed alterations in the duration of reflexive saccades, number of corrected antisaccades, and number of anticipated antisaccades compared with both control and NMHE groups; however, no significant differences were observed between MHE and early-MHE patients for these parameters (Fig 1A; Table 5).

For additional vertical antisaccade parameters including latency, latency of reflexive saccades, negative error, and its SD, significant impairments were observed only in patients with MHE compared with all other groups (Fig 1A; Table 5).

In the fixation task, patients with both early-MHE and MHE showed significant alterations in the number of saccades, the bivariate contour ellipse area (BCEA), and horizontal standard deviation (Ox) compared with both NMHE patients and controls (Fig 1C; Table 5).

Patients with MHE patients also exhibited additional abnormalities compared to control and NMHE patients, including increased biphasic square wave jerks, increased vertical standard deviation (Oy), altered horizontal centroid position (centroid x), and changes in microsaccade parameters, including amplitude, velocity and peak velocity (Fig 1C; Table 5). Differences between MHE and early-MHE groups were observed only for BCEA and horizontal centroid position (centroid x) (Fig 1C; Table 5).

To assess whether sex distribution or liver disease severity influenced eye movement outcomes, interaction analyses were performed, as shown in S6–S15 Tables. No significant interactions were found between sex and any eye movement variable (S6–S10 Tables). Regarding liver disease severity, measured by Child–Pugh and MELD scores, the highest number of interacting variables was observed in the horizontal and vertical smooth pursuit tasks, as well as in selected parameters of the vertical antisaccade task (Tables 4,5 and S13–S14 Tables). Horizontal antisaccades parameters did not show any significant interaction with liver disease severity scales (S14 Table).

### Analysis of the predictive capacity of cognitive impairment using eye movement parameters

To evaluate the ability of eye movement parameters to classify cirrhotic patients with mild cognitive alterations, univariate logistic regression analyses and ROC curve analyses were performed for all variables showing significant differences between the groups with cognitive alterations (early-MHE and MHE patients) compared with NMHE group. The predictive group was established as the set of patients classified with mild cognitive alterations (early-MHE + MHE patients). A large number of eye movement parameters showed significant predictive ability for early cognitive alterations (S16 and S17 Tables). However, only 12 parameters achieved an area under the ROC curve (AUROC) higher than 0.7 (Table 6).

The parameters with the highest discriminative performance are summarized in Table 6, including AUROC values, p values, optimal cut-off points, sensitivity, and specificity. These variables were mainly derived from antisaccade (horizontal and vertical) and fixation tasks.

### Multivariate models for the detection of early cognitive impairment

To develop a simple and time-efficient tool for the detection of early neurological alterations in cirrhotic patients, multivariate models were constructed using variables grouped by eye movement task. Separate models were built for vertical antisaccades (2 variables), fixation (3 variables), and horizontal antisaccades (6 variables), with the aim of identifying the most efficient single-task approach.

Due to methodological limitations of the eye-tracking system, variable based on variability of position error, such as standard deviation of negative error, may yield missing or null values when insufficient valid events are recorded. Therefore, this variable was excluded from the final horizontal antisaccade model, and it was composed of the six horizontal antisaccade variables showing the highest discriminative performance.

The different multivariate models evaluated are summarized in Table 7. The best predictive performance was obtained using the horizontal antisaccade model, which also provided the most practical implementation, as it relies on a single task while incorporating multiple informative variables.

**Table 7:** Predictive capability for early cognitive alterations in cirrhotic patients using several models based on eye-movement tests.

Model and variables	AUROC (95% CI)	p value	Sensitivity (%)	Specificity (%)
**Model: Horizontal antisaccades**				
Latency of reflexive saccades				
SD of Latency of reflexive saccades				
Duration of reflexive saccades	0.803 (0.711 - 0.896)	<0.0001	66.7	90.6
SD of Duration of reflexive saccades				
Incorrect antisaccades				
Anticipated antisaccades				
**Model: Vertical antisaccades**				
Latency of reflexive saccades	0.737 (0.609 - 0.866)	0.002	44.4	95.8
Positive error				
**Model: Fixation**				
Drift amplitude				
BCEA	0.762 (0.619 - 0.904)	0.0008	59.2	87.5
OX (Horizontal SD)				

AUROC, area under the receiver operating curve; BCEA, bivariate contour ellipse area SD, standard deviation

**Fig 2:**
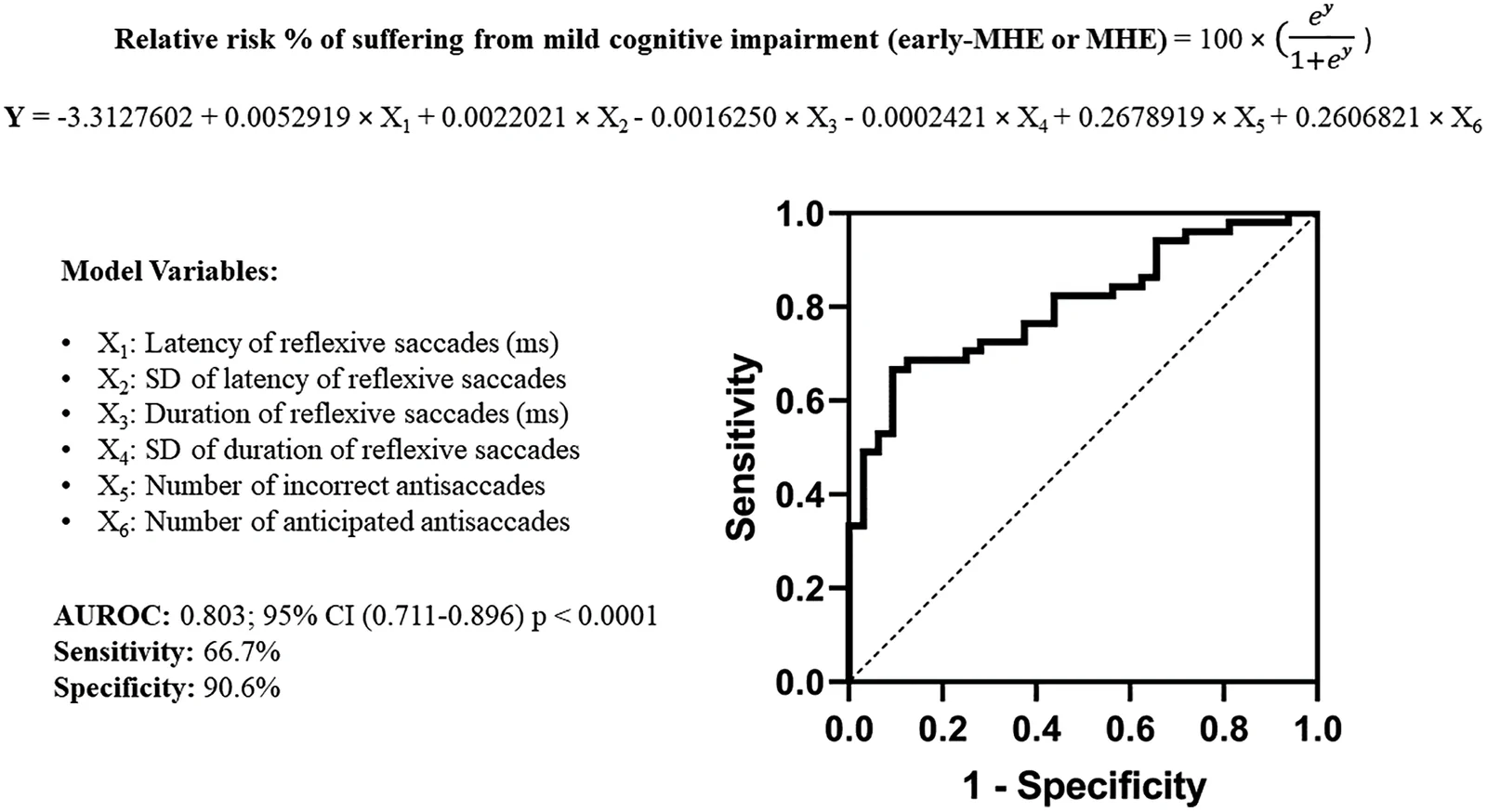
Formula and ROC curve of multivariate model to predict the patient risk of suffering mild cognitive alterations.

This model was used to estimate the patient risk of suffering mild cognitive alterations (early-MHE or MHE). The model achieved an AUROC value of 0.803 (0.711–0.896), with a sensitivity of 66.7% and a specificity of 90.6%, at an optimal cutoff of 65.66% relative risk (p < 0.001) (Table 7; Fig 2).

In the absence of an external validation cohort, internal validation of the model was performed using optimism-corrected bootstrapping. The procedure included 1000 bootstrapping iterations, yielding an optimism-corrected AUC value of 0.750 (95% CI: 0.646–0.858), similar to that obtained in the original model.

We tested whether potential confounding factors such as alcohol etiology or diabetes influenced the variables included in the predictive model. No differences were found between patients when classified according to cirrhosis etiology or diabetes status when comparing eye movement parameters of the predictive model (S18 and S19 Tables). Most of the differences between patient groups remained significant after these additional analyses, indicating that these factors do not interfere with the main findings of this study.

## Discussion

Minimal hepatic encephalopathy (MHE) represents a relevant social and healthcare burden, and its early identification is essential to prevent progression to overt hepatic encephalopathy (HE). Several studies have suggested that the Psychometric Hepatic Encephalopathy Score (PHES) although widely used, may lack sufficient sensitivity to detect subtle cognitive alterations in all patients with cirrhosis [14–17]. Therefore, additional approaches are needed to improve the detection of early cognitive dysfunction in this population. The objective of the present study was not to replace PHES as a reference tool, but rather to explore whether eye-movement analysis may identify cognitive alterations that are not detected by PHES alone and could therefore complement existing diagnostic strategies.

In a previous study, we demonstrated that patients with MHE present significant abnormalities in several eye-movement parameters and established the association between eye-movement alterations and cognitive impairment in cirrhosis [26]. The novelty of the present study lies in three main aspects. First, we identify and characterize a subgroup of cirrhotic patients who do not meet the PHES criteria for MHE but already exhibit subtle cognitive alterations (“early-MHE”). Second, we demonstrate for the first time that these patients also show specific eye-movement abnormalities, indicating that oculomotor alterations are present at earlier stages of cognitive dysfunction than previously recognized. Third, based on these findings, we develop and internally validate a predictive model based on eye-movement parameters that may be useful as a rapid screening tool for detecting early cognitive impairment in cirrhosis.

Early-MHE patients represented the 53.5% of individuals classified as not having MHE by PHES. These patients showed impaired performance across all psychometric tests compared with controls and NMHE patients, with a progressive worsening observed in patients with MHE, particularly in tasks assessing cognitive flexibility (Stroop incongruent task), mental processing speed (oral SDMT), and working memory (Digit Span forward). These findings support a continuum of cognitive impairment in cirrhosis ranging from NMHE to early-MHE and MHE.

**Fig 3:**
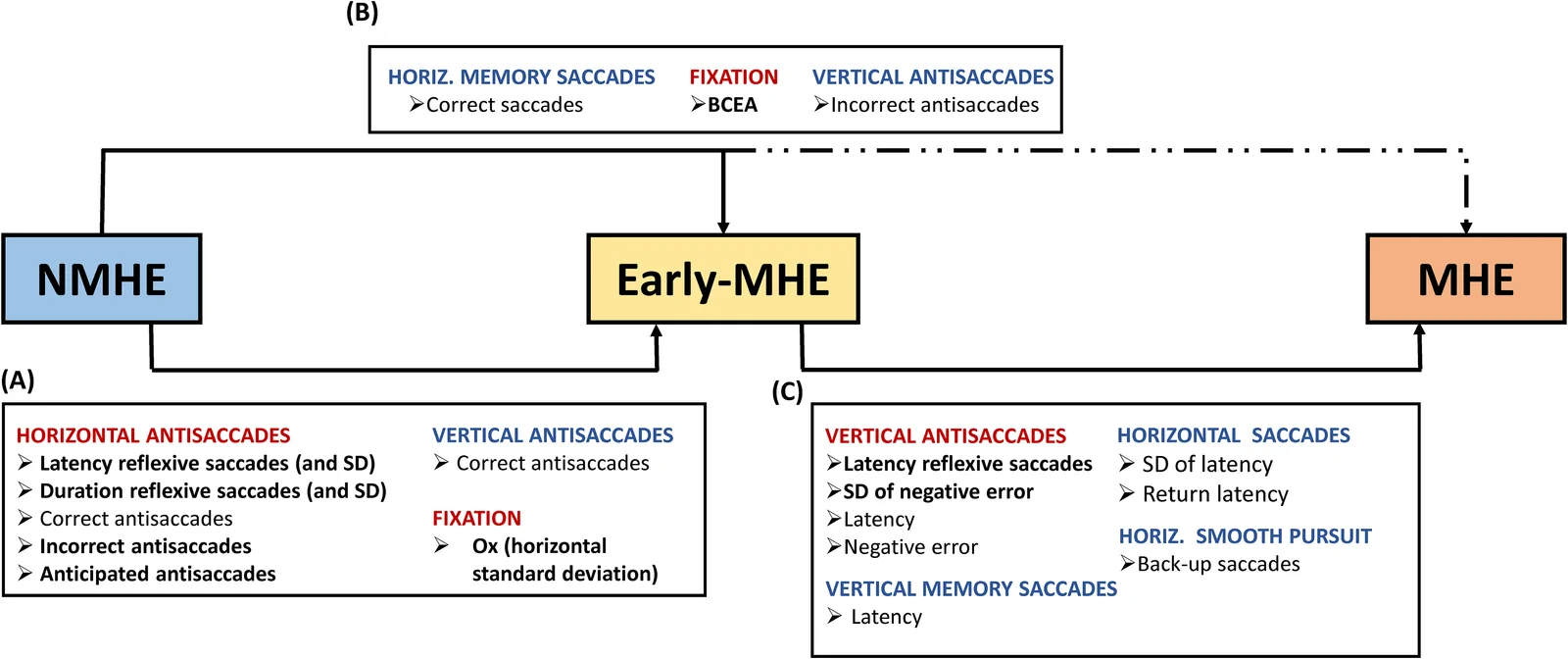
Cross-sectional comparison of eye movement alterations in cirrhotic patients according to cognitive status. (A) Eye movement parameters altered in early-MHE patients compared with controls and NMHE patients, and remaining altered in MHE patients. (B) Additional alterations present in early-MHE patients compared with healthy subjects and NMHE patients, and worsening in MHE patients. (C) Late alterations in the spectrum of cognitive impairment, exclusively characteristic of MHE patients. Variables with AUROC > 0.7 are shown in bold. Solid arrows indicate changes between cognitive stages, while dashed arrows indicate parameters that worsen in MHE compared with early-MHE. NMHE and MHE refer to patients without and with minimal hepatic encephalopathy, respectively; early-MHE refers to NMHE patients with early cognitive alterations; SD, standard deviation.

A similar graded pattern was also observed in eye movement parameters (Fig 3). Several oculomotor alterations appeared early in the spectrum, which were present in early-MHE and MHE patients, and were absent in NMHE patients (Fig 3A). Other abnormalities emerged in early-MHE patients and became more pronounced in MHE (Fig 3B), whereas some parameters were only altered in MHE (Fig 3C). This progressive pattern suggests a continuum of oculomotor dysfunction associated with worsening cognitive impairment.

Among all eye movement measures, parameters related to latency and duration of reflexive saccades, antisaccade performance, and measures of gaze precision and stability were the earliest and most consistently affected. Reflexive saccades are associated with parietal eye field function [31], and increased latency may reflect altered communication between the parietal eye field and the *superior colliculus* [32–36], a key structure in spatial attention and eye movement generation. Increased duration of reflexive saccades may also be related to reduced processing speed and attentional deficits, consistent with impairments observed in d2 and oral SDMT tests in early-MHE patients.

Gaze instability, reflected by increased Ox, BCEA, and vertical antisaccade errors, was observed in early-MHE and MHE patients. Ocular fixation is modulated by the interaction between the superior colliculus, *substantia nigra pars reticulata*, and *caudate nucleus* [37–39], suggesting that early cognitive impairment in cirrhosis may involve dysfunction within these basal ganglia–midbrain circuits. In addition, increased positive errors in antisaccade tasks may reflect impaired visuospatial transformation processes involving the lateral intraparietal sulcus and area 7a in the *precuneus* [40,41], consistent with previous findings in MHE [42–44].

Early-MHE patients also showed impaired antisaccade performance. Similar alterations have been reported in neurodegenerative disorders such as Parkinson’s and Alzheimer’s diseases, where they are associated with deficits in inhibitory control, attention, and working memory [19,45,46]. These cognitive domains were also impaired in early-MHE and MHE patients in the present study as shown by performance in the Stroop, d2, and Digit span tests.

ROC curve analyses demonstrated that several eye movement parameters have a good discriminatory ability for identifying patients with early cognitive alterations. To improve clinical applicability, multivariate models were developed, with the best performance achieved using variables from the horizontal antisaccade task. This model showed promising performance for estimating the risk of cognitive impairment (early-MHE + MHE), achieving an AUROC of 0.803, with 66.7% sensitivity and 90.6% specificity at a cut-off of 65.66%. Moreover, internal validation using optimism-corrected bootstrapping demonstrated internal stability and robustness of the model.

Given that the variables contributing to the final model are independent on sex distribution, diabetes, alcohol etiology or liver disease severity, the model developed discriminates between groups independently of these potential confounding factors. These findings suggest that the diagnostic performance of the model is not primarily driven by differences in sex distribution or liver disease severity across the study groups.

A limitation of this study lies on its cross-sectional design that, although the pattern of abnormalities observed across the study groups is compatible with increasing severity of cognitive alterations, the study design precludes conclusions regarding temporal progression or causal relationships. Prospective longitudinal studies would be required to determine whether the identified eye-movement abnormalities have prognostic value and whether their detection may ultimately translate into clinical benefit.

Although internal validation using optimism-corrected bootstrapping demonstrated good model stability, future external validation would represent the next essential step to confirm its robustness, generalizability, and clinical applicability.

Finally, the identification of the early-MHE subgroup and the associated eye-movement abnormalities should be considered exploratory until confirmed in independent prospective studies.

From a practical perspective, the antisaccade task requires only a few minutes to complete, suggesting that eye movement assessment may represent a rapid and objective tool for screening early cognitive alterations in cirrhotic patients. This characteristic may favor future clinical implementation compared with more complex approaches requiring multiple eye-movement tests. This approach could potentially help prioritize closer monitoring and earlier intervention.

## Conclusions

A proportion of patients with cirrhosis present subtle cognitive alterations that are not detected by the PHES battery, supporting a graded pattern of cognitive impairment associated with liver disease. This gradation is also reflected in eye movement abnormalities, which may provide sensitive markers of early neurological dysfunction in cirrhotic patients.

Among the evaluated eye movement tasks, antisaccade and fixation parameters, particularly those derived from the horizontal antisaccade task, showed the best ability to identify patients with early cognitive alterations. These findings suggest that eye movement analysis could represent a useful and objective approach for the detection of early cognitive alterations in cirrhosis. Further studies in independent cohorts are required to validate these findings and to determine their potential clinical utility.

## Supporting information

S1 Table Results of eye movement in horizontal and vertical visual saccades tests. (PDF)

S2 Table Results of eye movement in horizontal and vertical memory-guided saccades tests. (PDF)

S3 Table Results of eye movement in horizontal and vertical antisaccades tests. (PDF)

S4 Table Results of eye movement in horizontal, vertical and sinusoidal pursuit tests. (PDF)

S5 Table Results of eye movement in fixation test. (PDF)

S6 Table Analysis of interaction between sex distribution and eye-movement parameters: visual-guided test. (PDF)

S7 Table Analysis of interaction between sex distribution and eye-movement parameters: memory-guided test. (PDF)

S8 Table Analysis of interaction between sex distribution and eye-movement parameters: Smooth Pursuit tests. (PDF)

S9 Table Analysis of interaction between sex distribution and eye-movement parameters: Antisaccades test. (PDF)

S10 Table Analysis of interaction between sex distribution and eye-movement parameters: Fixation test. (PDF)

S11 Table Analysis of interaction between severity of liver damage and eye-movement parameters: visual-guided test. (PDF)

S12 Table Analysis of interaction between severity of liver damage and eye-movement parameters: memory-guided test. (PDF)

S13 Table Analysis of interaction between severity of liver damage and eye-movement parameters: Smooth Pursuit tests. (PDF)

S14 Table Analysis of interaction between severity of liver damage and eye-movement parameters: Antisaccades test. (PDF)

S15 Table Analysis of interaction between severity of liver damage and eye-movement parameters: Fixation test. (PDF)

S16 Table Diagnostic accuracy of eye movement variables to detect mild cognitive alterations in cirrhotic patients by univariate logistic regression analyses. (PDF)

S17 Table Diagnostic accuracy of eye movement variables to detect mild cognitive alterations in cirrhotic patients by ROC analyses. (PDF)

S18 Table Comparison of antisaccade parameters included in the model between patients with alcoholic etiology and with other etiologies, in the three groups of patients. (PDF)

S19 Table Comparison of antisaccade parameters included in the model between patients with and without diabetes mellitus in the three groups of patients. (PDF)
