# Supplementary material for: Eye movement analysis detects early neurological alterations in cirrhotic patients classified as not having minimal hepatic encephalopathy by the Psychometric Hepatic Encephalopathy Score
Source: PLoS One. 2026 Sep 15;21(9):e0358353. doi: 10.1371/journal.pone.0358353 (PMC13577538; doi:10.1371/journal.pone.0358353)
Supplement: Supplementary file 1 [file pone.0358353.s001.pdf]

Eye movement analysis detects early neurological alterations in cirrhotic patients classified as not having minimal hepatic encephalopathy by the Psychometric Hepatic Encephalopathy Score

Juan-José Gallego[¶], Franc Casanova-Ferrer[¶], Alessandra Fiorillo, Adrià López-Gramaje, Danna Campos, Amparo Urios, Javier Megías, Teresa San-Miguel, Montserrat Alcahuz-Griñán, Lucía Durbán, Elena Kosenko, Cecilia E. García-Cena, Carmina Montoliu*

[¶]These authors contributed equally to this work. *Corresponding author: Carmina Montoliu

S1 Table. Results of eye movement in horizontal and vertical visual saccades tests.

Variable	Control	NMHE	Early-MHE	MHE	Global FDR values
**Horizontal visual saccades**					
Latency (ms)	225.0 (26.3)	222.1 (34.1)	232.5 (36.6)	247.4 (60.7)*	ns
SD of latency	37.6 (20.4)	35.6 (29.5)	34.4 (17.5)	48.6 (51.0)$^{*/\alpha/\beta}$	ns
Gain	1.03 (0.08)	1.03 (0.04)	1.03 (0.05)	1.05 (0.07)	ns
SD of gain	0.07 (0.06)	0.06 (0.08)	0.08 (0.08)	0.08 (0.05)	ns
Peak of velocity (°/s)	399.9 (151.8)	341.8 (129.8)	354.2 (109.7)	322.5 (119.2)	ns
SD of peak of velocity	136.6 (63.1)	129.0 (70.8)	148.5 (52.5)	130.5 (53.0)§	ns
Positive error	0.56 (0.48)	0.49 (0.73)	0.53 (0.31)	0.79 (0.80)	ns
SD of positive error	0.31 (0.28)	0.28 (0.40)	0.33 (0.24)	0.49 (0.33)	ns
Negative error	-0.18 (0.23)	-0.31 (0.92)	-0.26 (0.42)	-0.33 (0.67)	ns
SD of negative error	0.19 (0.33)	0.19 (0.49)	0.19 (0.19)	0.18 (0.37)	ns
Blinks	2 (6)	1 (6)	2 (5)	4 (7)	ns
Number of anticipated antisaccades	0 (1)	0 (1)	0 (1)	1 (1)§	ns
Return latency (ms)	201.0 (33.6)	212.3 (23.8)	209.0 (33.4)	239.0 (47.4)$^{*/\beta/\S}$	ns
SD of return latency	32.43 (20.46)	34.79 (22.44)	33.98 (16.19)	41.75 (32.92)	ns
Return peak of velocity (°/s)	373.3 (138.3)	317.8 (64.2)	346.3 (120.5)§	315.1 (95.1)	ns
SD of return peak of velocity	161.8 (69.5)	148.9 (37.5)	173.8 (67.0)§	156.0 (49.1)	ns
**Vertical visual saccades**					
Latency (ms)	246.6 (33.0)	245.6 (45.6)	258.9 (36.2)*	275.6 (55.6)$^{*/\alpha}$	ns
SD of latency	44.6 (23.0)	38.2 (20.6)	43.3 (26.4)	45.6 (23.3)	ns
Gain	1.04 (0.08)	1.02 (0.08)	1.04 (0.09)	1.01 (0.10)	ns
SD of gain	0.11 (0.11)	0.13 (0.14)	0.11 (0.09)	0.16 (0.12)	ns
Peak of velocity (°/s)	272.0 (60.8)	257.9 (87.5)	249.8 (65.1)	234.4 (99.9)	ns
SD of peak of velocity	88.5 (45.7)	98.8 (60.7)	92.6 (44.7)	86.1 (48.3)	ns
Positive error	0.64 (0.46)	0.47 (0.62)	0.53 (0.45)	0.55 (0.68)	ns
SD of positive error	0.38 (0.40)	0.26 (0.32)	0.34 (0.33)	0.34 (0.26)	ns
Negative error	-0.27 (0.20)	-0.36 (0.68)	-0.28 (0.48)	-0.37 (0.58)	ns
SD of negative error	0.23 (0.30)	0.23 (0.30)	0.17 (0.34)	0.20 (0.26)	ns
Blinks	2 (2)	2 (4)	1 (4)	3 (5)	ns
Number of anticipated antisaccades	0 (0)	0 (2)	0 (1)	0 (1)	ns
Return latency (ms)	221.4 (39.4)	226.3 (39.5)	243.2 (45.2)*	261.1 (52.4)$^{***/\alpha}$	0.010
SD of return latency	29.6 (18.9)	34.0 (27.9)*	41.3 (30.3)**	42.1 (39.6)*	ns
Return peak of velocity (°/s)	256.5 (77.3)	255.0 (95.5)	248.1 (97.7)	231.3 (106.0)	ns
SD of return peak of velocity	91.3 (39.4)	104.6 (44.0)	83.0 (36.7)	80.7 (45.1)	ns

Values are expressed as median (IQR). Values significantly different from the control are indicated by an asterisk (*), from NMHE patients by α, and from early-MHE by β ($^{*/\alpha/\beta}$ $p < 0.05$; ** $p < 0.01$; *** $p < 0.001$). § Significant interaction between severity of liver damage (Child Pugh and/or MELD scores) and variable tested (see S11Table). NMHE, without minimal hepatic encephalopathy; MHE, minimal hepatic encephalopathy; SD, standard deviation.
