# Supplementary material for: Eye movement analysis detects early neurological alterations in cirrhotic patients classified as not having minimal hepatic encephalopathy by the Psychometric Hepatic Encephalopathy Score
Source: PLoS One. 2026 Sep 15;21(9):e0358353. doi: 10.1371/journal.pone.0358353 (PMC13577538; doi:10.1371/journal.pone.0358353)
Supplement: Supplementary file 2 [file pone.0358353.s002.pdf]

Eye movement analysis detects early neurological alterations in cirrhotic patients classified as not having minimal hepatic encephalopathy by the Psychometric Hepatic Encephalopathy Score

Juan-José Gallego[¶], Franc Casanova-Ferrer[¶], Alessandra Fiorillo, Adrià López-Gramaje, Danna Campos, Amparo Urios, Javier Megías, Teresa San-Miguel, Montserrat Alcahuz-Griñán, Lucía Durbán, Elena Kosenko, Cecilia E. García-Cena, Carmina Montoliu*

[¶]These authors contributed equally to this work *Corresponding author: Carmina Montoliu

S2 Table. Results of eye movement in horizontal and vertical memory-guided saccades tests.

Variable	Control	NMHE	Early-MHE	MHE	Global FDR values
**Horizontal memory-guided saccades**					
Latency (ms)	288.1 (87.9)	339.9 (136.5)*	318.2 (118.9)*	372.0 (168.4)**	ns
SD of latency	82.7 (85.0)	112.9 (114.8)	127.2 (123.3)*	127.4 (91.7)	ns
Gain	1.05 (0.13)	1.00 (0.12)	1.02 (0.19)	0.97 (0.19)*	ns
SD of gain	0.16 (0.11)	0.17 (0.10)	0.17 (0.16)	0.16 (0.22)	ns
Peak of velocity (º/s)	323.9 (140.3)	315.4 (124.5)	336.6 (156.0)	299.7 (90.2)	ns
SD of peak of velocity	136.2 (63.1)	127.5 (57.2)	140.2 (82.4)	119.0 (84.3)	ns
Positive error	1.63 (1.33)	1.48 (0.96)	1.68 (1.09)	1.38 (0.55)	ns
SD of positive error	0.72 (0.87)	0.86 (0.69)	1.11 (0.61)§	0.71 (0.45)	ns
Negative error	-0.78 (0.95)	-0.83 (1.10)	-1.18 (1.15)	-1.11 (1.75)	ns
SD of negative error	0.78 (0.97)	0.67 (0.88)	0.64 (0.58)	1.22 (1.12)	ns
Blinks	4.50 (8.75)	5.00 (9.25)	3.50 (10.25)	7.00 (17.25)	ns
Number of anticipated saccades	3 (5)	2 (3)	1 (3)	2 (3)	ns
Number of correct saccades	11 (2)	11 (4)	10 (7)*$^{/\alpha}$	4 (6)***$^{/\alpha\alpha\alpha/\beta/\S}$	<0.001
Return latency (ms)	285.3 (137.2)	285.9 (127.8)	311.1 (164.3)	325.9 (121.3)	ns
SD of return latency	91.4 (73.7)	91.9 (67.3)	93.3 (96.5)	93.3 (90.7)	ns
Return peak of velocity (º/ms)	364.9 (199.5)	311.4 (153.0)	346.4 (149.9)	319.1 (117.6)	ns
SD of return peak of velocity	170.2 (58.9)	161.2 (71.6)	161.6 (72.4)	134.3 (79.7)	ns
**Vertical memory-guided saccades**					
Latency (ms)	244.4 (81.2)	324.2 (155.9)**	296.6 (140.6)*	422.0 (248.0)***$^{/\alpha/\beta}$	0.003
SD of latency	51.45 (56.4)	101.1 (144.4)	94.9 (112.0)*	149.8 (129.6)	ns
Gain	1.02 (0.19)	0.98 (0.21)	0.97 (0.19)	1.01 (0.29)	ns
SD of gain	0.13 (0.10)	0.18 (0.17)	0.14 (0.12)	0.24 (0.28)	ns
Peak of velocity (º/s)	256.7 (70.7)	250.1 (83.9)	239.5 (115.3)§	243.5 (128.7)§	ns
SD of peak of velocity	101.1 (55.8)	95.4 (31.0)	83.9 (48.6)	60.1 (102.5)§	ns
Positive error	1.51 (0.98)	1.06 (1.11)	0.91 (0.71)	2.13 (1.02)β	ns
SD of positive error	0.76 (0.52)	0.73 (0.75)	0.56 (0.45)	1.52 (1.38)	ns
Negative error	-0.77 (0.90)	-1.19 (1.33)	-1.20 (1.13)	-1.08 (1.45)	ns
SD of negative error	0.35 (0.57)	0.58 (0.61)	0.59 (0.52)	0.36 (0.73)§	ns
Blinks	5 (6.5)	5 (7)	2 (9)	6 (12)	ns
Number of anticipated saccades	2 (3)	1 (3)	2 (3)	1 (1)	ns
Number of correct saccades	8 (1)	8 (2)	6 (6)**	2 (3)***$^{/\alpha\alpha\alpha/\beta\beta}$	<0.001
Return latency (ms)	241.0 (90.9)	271.2 (106.2)	286.4 (158.8)**	317.5 (143.2)*	ns
SD of return latency	70.5 (59.7)	59.1 (98.4)	89.1 (110.7)	130.0 (99.0)	ns
Return peak of velocity (º/ms)	254.6 (107.8)	238.4 (104.3)	200.9 (135.8)§	216.8 (139.8)§	ns
SD of return peak of velocity	113.9 (74.3)	104.0 (38.3)	87.13 (37.5)	111.0 (42.9)	ns

Values are expressed as median (IQR). Values significantly different from the control are indicated by an asterisk (*), from NMHE patients by α, and from early-MHE by β ($^{*/\alpha/\beta}$ $p < 0.05$; $^{**/\beta\beta}$ $p < 0.01$; $^{***/\alpha\alpha\alpha/\beta\beta}$ $p < 0.001$). § Significant interaction between severity of liver damage (Child Pugh and/or MELD scores) and variable tested (see S12Table). NMHE, without minimal hepatic encephalopathy; MHE, minimal hepatic encephalopathy; SD, standard deviation
