# Supplementary material for: Eye movement analysis detects early neurological alterations in cirrhotic patients classified as not having minimal hepatic encephalopathy by the Psychometric Hepatic Encephalopathy Score
Source: PLoS One. 2026 Sep 15;21(9):e0358353. doi: 10.1371/journal.pone.0358353 (PMC13577538; doi:10.1371/journal.pone.0358353)
Supplement: Supplementary file 3 [file pone.0358353.s003.pdf]

Eye movement analysis detects early neurological alterations in cirrhotic patients classified as not having minimal hepatic encephalopathy by the Psychometric Hepatic Encephalopathy Score

Juan-José Gallego¶, Franc Casanova-Ferrer¶, Alessandra Fiorillo, Adrià López-Gramaje, Danna Campos, Amparo Urios, Javier Megías, Teresa San-Miguel, Montserrat Alcahuz-Griñán, Lucía Durbán, Elena Kosenko, Cecilia E. García-Cena, Carmina Montoliu*

¶These authors contributed equally to this work. *Corresponding author: Carmina Montoliu

S3 Table. Results of eye movement in horizontal and vertical antisaccades tests.

Variable	Control	NMHE	Early-MHE	MHE	Global FDR values
**Horizontal antisaccades**					
Latency (ms)	387.1 (91.0)	391.0 (168.3)	493.8 (252.3)	480.0 (444.3)	ns
SD of latency	82.4 (69.7)	61.6 (66.8)	173.1 (153.3)*/α	81.3 (101.6)	0.050
Latency of reflexive saccades (ms)	550.5 (162.7)	551.0 (205.7)	673.1 (331.4)**/ααα	727.8 (359.8)***/ααα	<0.001
SD of latency of reflexive saccades	120.4 (68.9)	124.5 (100.0)	180.5 (191.3)*/αα	212.3 (91.7)**/αα	0.005
Duration of reflexive saccades (ms)	194.4 (90.0)	215.2 (186.3)	306.3 (273.0)**/αα	345.4 (285.8)***/ααα	<0.001
SD of duration of reflexive saccades	112.6 (72.8)	99.5 (103.4)	183.0 (150.1)*/αα	194.6 (90.7)**/αα	0.005
Positive error	2.09 (2.58)	2.08 (2.22)	3.54 (5.22)*	5.28 (4.53)**/αα	0.007
SD of positive error	0.909 (1.180)	1.680 (2.612)	2.366 (2.588)*	3.60 (3.07)***/α	0.003
Negative error	-2.11 (2.30)	-2.49 (2.12)	-4.03 (7.27)*	-4.65 (6.40)**/α	0.018
SD of negative error	1.62 (2.10)	1.49 (1.03)	3.14 (4.34)*/α	4.97 (6.96)**/αα	0.003
Blinks	6 (8)	2 (5)	2 (4)	5 (8)	ns
Number of correct antisaccades	3 (6)	3 (5)	1 (3)**/αα	0 (2)**/ααα	<0.001
Number of corrected antisaccades	5 (7)	6 (5)	5 (5)	4 (6)	ns
Number of incorrect antisaccades	0 (1)	0 (0)	1 (3)**/αα	3 (6)**/ααα	<0.001
Number of SO reflexive saccades	0 (0)	0 (0)	0 (0)	0 (1)α	ns
Number of anticipated antisaccades	0 (1)	0 (1)	1 (3)*/αα	1 (3)*/α	0.008
Return latency (ms)	320.4 (147.3)	362.3 (182.6)	399.4 (154.9)*	358.7 (168.4)	ns
SD of return latency	99.4 (55.6)	86.7 (89.6)	152.0 (126.8)*/α	157.7 (148.1)	0.022
Return peak of velocity (º/ms)	287.9 (150.5)	337.5 (104.9)	330.8 (141.4)	312.1 (112.4)	ns
SD of return peak of velocity	165.6 (78.7)	157.6 (105.7)	157.9 (66.1)	170.8 (78.3)	ns
**Vertical antisaccades**					
Latency (ms)	393.1 (67.9)	397.6 (140.8)	430.0 (167.3)	583.9 (242.1)**/αα/β	0.012
SD of latency	72.3 (87.1)	65.0 (91.1)	124.4 (150.7)	90.47 (126.9)	ns
Latency of reflexive saccades (ms)	527.4 (104.9)	523.6 (201.0)	580 (160.3)	959.2 (521.0)***/ααα/ββ	0.001
SD of latency of reflexive saccades	95.5 (74.0)	89.1 (74.4)	119.9 (99.4)	94.76 (147.0)	ns
Duration of reflexive saccades (ms)	173.6 (63.4)	200.8 (108.8)	222.2 (125.3)	271.9 (359.8)*/α	ns
SD of duration of reflexive saccades	77.1 (88.3)	98.8 (76.5)	101.3 (77.2)	98.53 (93.7)	ns
Positive error	1.59 (1.43)	1.54 (1.82)	2.09 (2.99)*/α	3.11 (1.96)**/αα/§	0.010
SD of positive error	0.88 (0.97)	1.18 (1.46)	1.04 (1.11)	2.09 (1.21)*/§	ns
Negative error	-2.51 (2.40)	-2.35 (2.75)	-3.67 (3.87)	-5.47 (6.05)**/αα/β	0.026
SD of negative error	1.45 (1.77)	1.40 (2.04)	1.26 (2.01)	4.34 (5.04)*/α/β	ns
Blinks	3 (6)	3 (4)	2 (3)	4 (4)	ns
Number of correct antisaccades	2 (4)	2 (2)	1 (2)*/α/§	1 (2)*/α/§	0.016
Number of corrected antisaccades	3 (4)	4 (4)	3 (4)§	2 (3)*/α	ns
Number of incorrect antisaccades	0 (1)	0 (1)	1 (2)*/α	1 (3)***/ααα/β	0.002
Number of SO reflexive saccades	0 (0)	0 (0)	0 (0)	0 (1)	ns
Number of anticipated antisaccades	0 (1)	0 (1)	0 (2)	1 (2)**/α	0.028
Return latency (ms)	336.8 (165.3)	361.0 (151.2)	373.8 (146.3)	354.7 (195.3)§	ns
SD of return latency	83.6 (66.4)	87.1 (112.5)	90.0 (95.7)	106.7 (101.4)§	ns
Return peak of velocity (º/ms)	239.7 (102.3)	181.7 (87.9)	221.5 (67.0)	223.8 (98.7)§	ns
SD of return peak of velocity	112.9 (60.2)	93.4 (51.7)	98.2 (60.1)	145.1 (57.3)α	ns

Values are expressed as median (IQR). Values significantly different from the control are indicated by an asterisk (*), from NMHE patients by α, and from early-MHE by β ($^{*/\alpha/\beta}$ $p < 0.05$; $^{**/\alpha\alpha/\beta\beta}$ $p < 0.01$; $^{***/\alpha\alpha\alpha}$ $p < 0.001$). § Significant interaction between severity of liver damage (Child Pugh and/or MELD scores) and variable tested (see S14Table). NMHE, without minimal hepatic encephalopathy; MHE, minimal hepatic encephalopathy; SD, standard deviation; SO, second order.
