# Supplementary material for: Eye movement analysis detects early neurological alterations in cirrhotic patients classified as not having minimal hepatic encephalopathy by the Psychometric Hepatic Encephalopathy Score
Source: PLoS One. 2026 Sep 15;21(9):e0358353. doi: 10.1371/journal.pone.0358353 (PMC13577538; doi:10.1371/journal.pone.0358353)
Supplement: Supplementary file 4 [file pone.0358353.s004.pdf]

Eye movement analysis detects early neurological alterations in cirrhotic patients classified as not having minimal hepatic encephalopathy by the Psychometric Hepatic Encephalopathy Score

Juan-José Gallego¶, Franc Casanova-Ferrer¶, Alessandra Fiorillo, Adrià López-Gramaje, Danna Campos, Amparo Urios, Javier Megías, Teresa San-Miguel, Montserrat Alcahuz-Griñán, Lucía Durbán, Elena Kosenko, Cecilia E. García-Cena, Carmina Montoliu*

¶These authors contributed equally to this work. *Corresponding author: Carmina Montoliu

S4 Table. Results of eye movement in horizontal, vertical and sinusoidal pursuit tests.

Variable	Control	NMHE	Early-MHE	MHE	Global FDR values
**Horizontal smooth pursuit**					
Blinks	2 (5)	1 (3)	1 (3)	3 (4)	ns
Cath-up saccades	20 (14)	20 (9)	21 (9)§	25 (11)§	ns
Back-up saccades	0 (0)	0 (1)	0 (1)	1 (2)**$^{/\alpha/\beta}$	0.048
Square wave jerks	3 (4)	4 (6)	4 (5)	5 (4)	ns
Pursuit time (%)	92.5 (5.5)	92.1 (5.5)	91.1 (6.1)§	88.3 (4.5)*$^{/\alpha/§}$	ns
Latency (ms)	305.3 (53.1)	295.9 (57.1)	314.0 (118.8)	304.3 (211.4)§	ns
Total mean squared error of position	1.98 (1.51)	2.32 (1.54)	2.59 (2.68)*$^{/ §}$	3.19 (3.56)*$^{/\alpha/§}$	0.048
Pursuit mean squared error of position	1.96 (1.41)	2.25 (1.56)	2.53 (2.61)*	2.99 (3.47)*$^{/\alpha}$	0.048
Gain	0.87 (0.17)	0.89 (0.29)	0.79 (0.19)*	0.73 (0.14)**$^{/\alpha\alpha/§}$	0.024
Pursuit mean squared error of velocity	8.70 (2.47)	8.22 (1.90)	9.26 (2.46)	8.99 (1.91)	ns
**Vertical smooth pursuit**					
Blinks	2 (5)	2 (3)	1 (4)	3 (8)	ns
Cath-up saccades	13 (12)	14 (7)	15 (4)§	18 (6)*$^{/\alpha/§}$	0.041
Back-up saccades	0 (1)	0 (2)	0.5 (2)	1 (3)	ns
Square wave jerks	1 (2.5)	2 (4)	2 (5)*	3 (3)*	0.041
Pursuit time (%)	93.1 (5.6)	92.2 (4.9)	91.8 (6.9)§	90.0 (9.0)*$^{/\alpha/§}$	ns
Latency (ms)	396.0 (208.4)	359.8 (161.6)	364.0 (192.1)	444.1 (235.4)	ns
Total mean squared error of position	1.50 (1.64)	1.61 (1.98)	1.86 (2.45)	2.72 (2.02)**$^{/\alpha\alpha}$	0.016
Pursuit mean squared error of position	1.44 (1.49)	1.50 (1.66)	1.78 (1.97)	2.63 (1.9)**$^{/\alpha\alpha}$	0.016
Gain	0.83 (0.23)	0.83 (0.20)	0.79 (0.20)§	0.70 (0.18)**$^{/\alpha/§}$	0.033
Pursuit mean squared error of velocity	5.97 (2.06)	5.95 (2.21)	6.27 (2.39)§	7.26 (3.48)§	ns
**Sinusoidal smooth pursuit**					
Blinks	1 (3)	2 (4)	2 (5)	2 (3)	ns
Cath-up saccades	19 (14)	16 (11)	16 (8)§	21 (6)$^{\alpha/§}$	ns
Back-up saccades	0 (1)	0 (1)	0 (2)	0 (2)	ns
Square wave jerks	1 (5)	2 (5)	3 (6)	4 (4)	ns
Pursuit time (%)	93.3 (7.1)	93.4 (6.2)	91.4 (5.6)	90.2 (6.9)	ns
Latency (ms)	259.7 (36.0)	264.2 (51.6)	272.0 (52.7)	308.3 (77.7)*	ns
Total mean squared error of position	1.65 (2.80)	2.24 (2.21)	2.52 (2.02)	3.86 (2.94)*$^{/\alpha}$	ns
Pursuit mean squared error of position	1.63 (2.69)	2.11 (2.12)	2.35 (1.82)	3.72 (2.83)*$^{/\alpha}$	ns
Gain	0.86 (0.22)	0.83 (0.36)	0.76 (0.33)	0.67 (0.30)$^{\alpha}$	ns
Pursuit mean squared error of velocity	8.80 (1.88)	8.38 (1.97)	9.40 (3.19)	9.46 (2.17)	ns

Values are expressed as median (IQR). Values significantly different from the control are indicated by an asterisk (*), from NMHE patients by α, and from early-MHE by β ($^{*/\alpha/\beta}$ $p < 0.05$; $^{**/\alpha\alpha}$ $p < 0.01$). § Significant interaction between severity of liver damage (Child Pugh and/or MELD scores) and variable tested (see S14 Table). NMHE, without minimal hepatic encephalopathy; MHE, minimal hepatic encephalopathy; SD, standard deviation.
