# Supplementary material for: Eye movement analysis detects early neurological alterations in cirrhotic patients classified as not having minimal hepatic encephalopathy by the Psychometric Hepatic Encephalopathy Score
Source: PLoS One. 2026 Sep 15;21(9):e0358353. doi: 10.1371/journal.pone.0358353 (PMC13577538; doi:10.1371/journal.pone.0358353)
Supplement: Supplementary file 5 [file pone.0358353.s005.pdf]

Eye movement analysis detects early neurological alterations in cirrhotic patients classified as not having minimal hepatic encephalopathy by the Psychometric Hepatic Encephalopathy Score

Juan-José Gallego[¶], Franc Casanova-Ferrer[¶], Alessandra Fiorillo, Adrià López-Gramaje, Danna Campos, Amparo Urios, Javier Megías, Teresa San-Miguel, Montserrat Alcahuz-Griñán, Lucía Durbán, Elena Kosenko, Cecilia E. García-Cena, Carmina Montoliu*

[¶]These authors contributed equally to this work. *Corresponding author: Carmina Montoliu

S5 Table. Results of eye movement in fixation test.

Variable	Control	NMHE	Early-MHE	MHE	Global FDR values
			**Fixation**		
Blinks	3 (4)	2 (5)	2 (4)	5 (5)α	ns
Saccades	2 (3)	1 (4)	3 (5)$^{*/\alpha/\S}$	4 (6)*/α	ns
Microsaccades	5 (7)	6 (9)	5 (10)§	8 (13)	ns
Number of drifts	1 (2)	1 (4)	1 (2)	2 (6)§	ns
Monophasic- square wave jerks	0 (3)	0 (2)	1 (3)	2 (3)	ns
Biphasic- square wave jerks	0 (0)	0 (1)	0 (2)	1 (3)*/α	ns
Distractions	0 (0)	0 (0)	0 (0)	0 (1)	ns
BCEA (º)	0.44 (0.46)	0.31 (0.69)	1.04 (2.28)$^{*/\alpha\alpha}$	1.39 (2.50)$^{**/\alpha\alpha\alpha/\beta}$	0.003
Ox (horizontal standard deviation) (º)	0.27 (0.12)	0.23 (0.15)	0.39 (0.81)$^{**/\alpha\alpha}$	0.47 (0.50)$^{**/\alpha\alpha}$	0.006
Oy (vertical standard deviation) (º)	0.22 (0.19)	0.22 (0.32)	0.35 (0.70)	0.43 (0.37)$^{*/\alpha}$	ns
Centroid x (horizontal) (º)	0.08 (0.26)	0.26 (0.43)	0.24 (0.46)*	0.12 (0.20)$^{*/\alpha/\beta}$	ns
Centroid y (vertical) (º)	0.26 (0.33)	0.40 (0.46)	0.54 (0.62)	0.72 (0.78)*	ns
Microsaccades amplitude (º)	0.34 (0.16)	0.36 (0.22)	0.42 (0.18)$^{*/\S}$	0.44 (0.10)$^{**/\alpha}$	0.046
SD of microsaccades amplitude	0.12 (0.10)	0.17 (0.15)	0.17 (0.12)	0.18 (0.07)	ns
Velocity of microsaccades (º/s)	13.8 (6.1)	14.4 (6.6)	14.5 (3.16)	16.3 (2.67)$^{*/\alpha}$	ns
SD of velocity of microsaccades	3.02 (2.72)	3.77 (3.34)	2.91 (2.80)	3.07 (2.07)	ns
Peak velocity-microsaccades (º/s)	24.5 (10.9)	24.5 (13.5)	26.8 (8.4)	28.8 (5.9)$^{*/\alpha}$	ns
SD of peak velocity-microsaccades	4.48 (5.42)	8.05 (8.05)	6.06 (7.57)	7.98 (5.77)	ns
Drift amplitude (º)	0.19 (0.21)	0.14 (0.13)	0.24 (0.14)	0.29 (0.11)$^{\alpha\alpha}$	ns
SD of drift amplitude	0.19 (0.21)	0.10 (0.05)	0.11 (0.10)	0.17 (0.09)	ns
Drift velocity (º/s)	2.58 (3.00)	2.34 (1.81)	2.18 (1.53)§	3.12 (2.58)	ns
SD of drift velocity	1.16 (1.67)	0.94 (0.99)	0.79 (1.19)	0.71 (1.01)	ns
Peak of velocity-drift (º/s)	10.7 (7.71)	7.85 (5.84)	7.81 (6.39)	9.56 (4.90)	ns
SD of peak of velocity-drift	2.60 (3.54)	3.90 (2.72)	3.39 (3.02)	3.75 (2.61)	ns
Square wave jerks—amplitude (º)	0.69 (0.35)	0.54 (0.39)	0.83 (0.79)	0.77 (0.42)	ns
SD of square wave jerks—amplitude	0.35 (0.36)	0.31 (0.34)	0.45 (0.50)	0.45 (0.46)	ns
Square wave jerks—time (ms)	226.6 (181.9)	298.0 (283.4)	237.3 (126.4)§	286.4 (93.5)	ns
SD of square wave jerks—time	173.8 (87.1)	188.4 (220.1)	113.6 (94.8)	168.3 (116.9)	ns

Values are expressed as median (IQR). Values significantly different from the control are indicated by an asterisk (*), from NMHE patients by α, and from early-MHE by β ($^{*/\alpha/\beta}$ $p < 0.05$; $^{**/\alpha\alpha}$ $p < 0.01$; $^{\alpha\alpha\alpha}$ $p < 0.001$). § Significant interaction between severity of liver damage (Child Pugh and/or MELD scores) and variable tested (see S15Table). NMHE, without minimal hepatic encephalopathy; MHE, minimal hepatic encephalopathy; SD, standard deviation; BCEA, bivariate contour ellipse area.
