# Supplementary material for: Eye movement analysis detects early neurological alterations in cirrhotic patients classified as not having minimal hepatic encephalopathy by the Psychometric Hepatic Encephalopathy Score
Source: PLoS One. 2026 Sep 15;21(9):e0358353. doi: 10.1371/journal.pone.0358353 (PMC13577538; doi:10.1371/journal.pone.0358353)
Supplement: Supplementary file 6 [file pone.0358353.s006.pdf]

Eye movement analysis detects early neurological alterations in cirrhotic patients classified as not having minimal hepatic encephalopathy by the Psychometric Hepatic Encephalopathy Score

Juan-José Gallego¶, Franc Casanova-Ferrer¶, Alessandra Fiorillo, Adrià López-Gramaje, Danna Campos, Amparo Urios, Javier Megías, Teresa San-Miguel, Montserrat Alcahuz-Griñán, Lucía Durbán, Elena Kosenko, Cecilia E. García-Cena, Carmina Montoliu*

¶These authors contributed equally to this work. *Corresponding author: Carmina Montoliu

S6 Table. Analysis of interaction between sex distribution and eye-movement parameters: visual-guided test.

	Visual-guided test (horizontal)			Visual-guided test (vertical)		
**Variable**	**Sex x NMHE**	**Sex x Early-MHE**	**Sex x MHE**	**Sex x NMHE**	**Sex x Early-MHE**	**Sex x MHE**
Latency (ms)	0.9920	0.3108	0.1744	0.8566	0.4042	0.0618
Standard Deviation of Latency	0.7668	0.0951	0.8874	0.9806	0.0762	0.7945
Gain	0.5095	0.4796	0.2690	0.8556	0.9494	0.6826
Standard Deviation of Gain	0.6163	0.7527	0.7102	0.4561	0.2508	0.7555
Peak of velocity (º/ms)	0.9061	0.636	0.9917	0.9086	0.6007	0.8281
Standard Deviation of Peak of Velocity	0.4883	0.2375	0.5208	0.9417	0.2204	0.5568
Positive Error	0.2012	0.8434	0.1332	0.0801	0.9221	0.9911
Standard Deviation of Positive Error	0.6789	0.7846	0.0991	0.1181	0.4457	0.8053
Negative Error	0.3706	0.5353	0.6355	0.5490	0.6576	0.4176
Standard Deviation of Negative Error	0.7343	0.5187	0.0904	0.7734	0.8364	0.9838
Blinks	0.7964	0.1994	0.5549	0.5110	0.8242	0.4880
Anticipated Saccades	0.9669	0.8510	0.1346	0.7343	0.2043	0.2376
Return latency (ms)	0.0828	0.9179	0.7765	0.4421	0.7568	0.4227
Standard Deviation of Return Latency	0.1465	0.9447	0.4336	0.1346	0.9525	0.5346
Return Peak of Velocity (º/ms)	0.9686	0.5006	0.6634	0.5738	0.5442	0.7749
SD of Return Peak of Velocity	0.6003	0.8753	0.7455	0.9020	0.6971	0.4791

P values for interaction between sex distribution and eye-movement parameters are shown, taking control group as reference. NMHE, MHE, patients without or with minimal hepatic encephalopathy, respectively; SD, Standard Deviation
