# Supplementary material for: Eye movement analysis detects early neurological alterations in cirrhotic patients classified as not having minimal hepatic encephalopathy by the Psychometric Hepatic Encephalopathy Score
Source: PLoS One. 2026 Sep 15;21(9):e0358353. doi: 10.1371/journal.pone.0358353 (PMC13577538; doi:10.1371/journal.pone.0358353)
Supplement: Supplementary file 7 [file pone.0358353.s007.pdf]

Eye movement analysis detects early neurological alterations in cirrhotic patients classified as not having minimal hepatic encephalopathy by the Psychometric Hepatic Encephalopathy Score

Juan-José Gallego¶, Franc Casanova-Ferrer¶, Alessandra Fiorillo, Adrià López-Gramaje, Danna Campos, Amparo Urios, Javier Megías, Teresa San-Miguel, Montserrat Alcahuz-Griñán, Lucía Durbán, Elena Kosenko, Cecilia E. García-Cena, Carmina Montoliu*

¶These authors contributed equally to this work. *Corresponding author: Carmina Montoliu

S7 Table. Analysis of interaction between sex distribution and eye-movement parameters: memory-guided test.

	Memory-guided test (horizontal)			Memory-guided test (vertical)		
**Variable**	**Sex x NMHE**	**Sex x Early-MHE**	**Sex x MHE**	**Sex x NMHE**	**Sex x Early-MHE**	**Sex x MHE**
Latency (ms)	0.4882	0.8740	0.3677	0.5469	0.4585	0.7162
Standard Deviation of Latency	0.1703	0.5457	0.1137	0.8616	0.2175	0.1817
Gain	0.5726	0.879	0.5829	0.1498	0.4947	0.1009
Standard Deviation of Gain	0.5066	0.5238	0.1308	0.4432	0.1396	0.4276
Peak of velocity (º/ms)	0.7823	0.4938	0.7291	0.8257	0.7244	0.4647
Standard Deviation of Peak of Velocity	0.8853	0.6171	0.4214	0.2387	0.6379	0.6724
Positive Errors	0.764	0.5049	0.8745	0.0753	0.2874	0.5679
Standard Deviation of Positive Errors	0.5401	0.6583	0.4829	0.3477	0.8448	0.2113
Negative Errors	0.9278	0.6542	0.0593	0.8548	0.8328	0.2314
Standard Deviation of Negative Errors	0.2080	0.4713	0.9712	0.1261	0.0828	0.0625
Blinks	0.5303	0.0883	0.7580	0.4640	0.1426	0.7822
Anticipated Saccades	0.4703	0.0543	0.0762	0.7573	0.5181	0.9309
Correct saccades	0.8455	0.646	0.3032	0.4482	0.6642	0.2684
Return latency (ms)	0.1117	0.5289	0.8657	0.7490	0.7111	0.7165
Standard Deviation of Return Latency	0.0957	0.2059	0.0948	0.8749	0.1142	0.4367
Return Peak of Velocity (º/ms)	0.5002	0.8495	0.4458	0.1221	0.3549	0.6459
Standard Deviation of Return Peak of Velocity	0.3401	0.2151	0.6023	0.0804	0.5414	0.3705

P values for interaction between sex distribution and eye-movement parameters are shown, taking control group as reference. NMHE, MHE, patients without or with minimal hepatic encephalopathy, respectively; SD, Standard Deviation
