# Supplementary material for: Eye movement analysis detects early neurological alterations in cirrhotic patients classified as not having minimal hepatic encephalopathy by the Psychometric Hepatic Encephalopathy Score
Source: PLoS One. 2026 Sep 15;21(9):e0358353. doi: 10.1371/journal.pone.0358353 (PMC13577538; doi:10.1371/journal.pone.0358353)
Supplement: Supplementary file 8 [file pone.0358353.s008.pdf]

Eye movement analysis detects early neurological alterations in cirrhotic patients classified as not having minimal hepatic encephalopathy by the Psychometric Hepatic Encephalopathy Score

Juan-José Gallego¶, Franc Casanova-Ferrer¶, Alessandra Fiorillo, Adrià López-Gramaje, Danna Campos, Amparo Urios, Javier Megías, Teresa San-Miguel, Montserrat Alcahuz-Griñán, Lucía Durbán, Elena Kosenko, Cecilia E. García-Cena, Carmina Montoliu*

¶These authors contributed equally to this work. *Corresponding author: Carmina Montoliu

S8 Table. Analysis of interaction between sex distribution and eye-movement parameters: Smooth Pursuit tests.

	Smooth pursuit test (Horizontal)			Smooth pursuit test (Vertical)			Smooth pursuit test (Sinusoidal)		
**Variable**	**Sex x NMHE**	**Sex x Early-MHE**	**Sex x MHE**	**Sex x NMHE**	**Sex x Early-MHE**	**Sex x MHE**	**Sex x NMHE**	**Sex x Early-MHE**	**Sex x MHE**
Blinks	0.1825	0.3386	0.4262	0.7194	0.5713	0.9281	0.5823	0.2584	0.2971
Catch-up saccades	0.6006	0.4265	0.1835	0.2161	0.2943	0.2287	0.3722	0.7440	0.3451
Back-up saccades	0.7333	0.7502	0.3542	0.8133	0.7136	0.4800	0.9337	0.5150	0.8559
Square Wave Jerks	0.0925	0.2770	0.7661	0.8539	0.4852	0.3807	0.0574	0.1089	0.4005
Pursuit Time (%)	0.0800	0.3707	0.4925	0.7312	0.6099	0.6658	0.7956	0.4317	0.5543
Latency (ms)	0.8368	0.7964	0.1014	0.9648	0.0613	0.1640	0.6451	0.8377	0.8180
Total Mean Squared Error of Position	0.2475	0.6491	0.8202	0.9054	0.3314	0.2828	0.1908	0.2255	0.9574
Pursuit Mean Squared Error of Position	0.2379	0.7046	0.7941	0.8991	0.3439	0.3057	0.2185	0.2575	0.9516
Gain	0.4879	0.2712	0.9903	0.7087	0.5495	0.8241	0.9983	0.3163	0.4865
Pursuit Mean Squared Error of Velocity	0.5847	0.6926	0.7023	0.5076	0.7443	0.3356	0.5753	0.5476	0.9469

P values for interaction between sex distribution and eye-movement parameters are shown, taking control group as reference. NMHE, MHE, patients without or with minimal hepatic encephalopathy, respectively.
