# Supplementary material for: Eye movement analysis detects early neurological alterations in cirrhotic patients classified as not having minimal hepatic encephalopathy by the Psychometric Hepatic Encephalopathy Score
Source: PLoS One. 2026 Sep 15;21(9):e0358353. doi: 10.1371/journal.pone.0358353 (PMC13577538; doi:10.1371/journal.pone.0358353)
Supplement: Supplementary file 9 [file pone.0358353.s009.pdf]

Eye movement analysis detects early neurological alterations in cirrhotic patients classified as not having minimal hepatic encephalopathy by the Psychometric Hepatic Encephalopathy Score

Juan-José Gallego¶, Franc Casanova-Ferrer¶, Alessandra Fiorillo, Adrià López-Gramaje, Danna Campos, Amparo Urios, Javier Megías, Teresa San-Miguel, Montserrat Alcahuz-Griñán, Lucía Durbán, Elena Kosenko, Cecilia E. García-Cena, Carmina Montoliu*

¶These authors contributed equally to this work. *Corresponding author: Carmina Montoliu

S9 Table. Analysis of interaction between sex distribution and eye-movement parameters: Antisaccades test.

	Antisaccades test (horizontal)			Antisaccades test (vertical)		
**Variable**	**Sex x NMHE**	**Sex x Early-MHE**	**Sex x MHE**	**Sex x NMHE**	**Sex x Early-MHE**	**Sex x MHE**
Latency (ms)	0.9275	0.5803	0.3812	0.7202	0.6631	0.1935
Standard Deviation of Latency	0.8404	0.3652	0.9817	0.7402	0.6559	0.7787
Latency of reflexive saccades (ms)	0.7073	0.5427	0.3067	0.7006	0.9664	0.6049
SD of Latency of reflexive saccades	0.5121	0.6937	0.7903	0.7927	0.6911	0.4490
Duration of reflexive saccades (ms)	0.8970	0.5123	0.5789	0.4056	0.9391	0.8786
SD of Duration of reflexive saccades	0.3144	0.5544	0.6557	0.9291	0.3409	0.0575
Positive Errors	0.7136	0.6248	0.6759	0.5419	0.8718	0.9192
SD of Positive Errors	0.9666	0.7331	0.3257	0.5284	0.7565	0.694
Negative Errors	0.4224	0.2656	0.2872	0.5407	0.5284	0.2897
SD of Negative Errors	0.5396	0.2345	0.3545	0.8685	0.7328	0.5587
Blinks	0.8815	0.9833	0.0645	0.9879	0.6733	0.127
Number of correct antisaccades	0.9735	0.6103	0.8224	0.1903	0.4746	0.4837
Number of corrected antisaccades	0.3729	0.8001	0.9155	0.4073	0.2958	0.6734
Number of incorrect antisaccades	0.6972	0.6564	0.2678	0.9384	0.4634	0.1599
Number of second order reflexive saccades	0.3132	0.1546	0.8242	0.9343	0.6961	0.5106
Number of Anticipated Saccades	0.3606	0.8125	0.3275	0.8634	0.2384	0.5330
Return latency (ms)	0.1848	0.1005	0.4494	0.3872	0.2572	0.2881
SD of Return Latency	0.9624	0.2702	0.6162	0.3712	0.0742	0.9744
Return Peak of velocity (°/ms)	0.7777	0.8195	0.5625	0.9570	0.4408	0.2369
SD of Return Peak of Velocity	0.9840	0.4133	0.8345	0.1775	0.9591	0.5205

P values for interaction between sex distribution and eye-movement parameters are shown, taking control group as reference. NMHE, MHE, patients without or with minimal hepatic encephalopathy, respectively; SD, Standard Deviation
