# Supplementary material for: Eye movement analysis detects early neurological alterations in cirrhotic patients classified as not having minimal hepatic encephalopathy by the Psychometric Hepatic Encephalopathy Score
Source: PLoS One. 2026 Sep 15;21(9):e0358353. doi: 10.1371/journal.pone.0358353 (PMC13577538; doi:10.1371/journal.pone.0358353)
Supplement: Supplementary file 10 [file pone.0358353.s010.pdf]

Eye movement analysis detects early neurological alterations in cirrhotic patients classified as not having minimal hepatic encephalopathy by the Psychometric Hepatic Encephalopathy Score

Juan-José Gallego¶, Franc Casanova-Ferrer¶, Alessandra Fiorillo, Adrià López-Gramaje, Danna Campos, Amparo Urios, Javier Megías, Teresa San-Miguel, Montserrat Alcahuz-Griñán, Lucía Durbán, Elena Kosenko, Cecilia E. García-Cena, Carmina Montoliu*

¶These authors contributed equally to this work. *Corresponding author: Carmina Montoliu

S10 Table. Analysis of interaction between sex distribution and eye-movement parameters: Fixation test.

Variable	Sex x NMHE	Sex x Early-MHE	Sex x MHE
Blinks	0.1599	0.5791	0.1446
Saccades	0.3319	0.286	0.5115
Microsaccades	0.5648	0.6035	0.9925
Number of Drifts	0.7209	0.8781	0.9369
Monophasic- Square Wave Jerks	0.3502	0.2298	0.9942
Biphasic- Square Wave Jerks	0.3114	0.3521	0.3502
Distractions	0.9659	0.9338	0.1636
BCEA (º)	0.8367	0.9907	0.1181
Ox (Horizontal Standard Deviation) (º)	0.8717	0.8979	0.1994
Oy (Vertical Standard Deviation) (º)	0.9307	0.9992	0.2256
Absolute Centroid X (Horizontal) (º)	0.8569	0.7008	0.2795
Absolute Centroid Y (Vertical) (º)	0.5839	0.6294	0.3421
Microsaccades Amplitude (º)	0.3557	0.0550	0.0560
Standard Deviation of Microsaccades Amplitude	0.3935	0.2468	0.4409
Velocity of Microsaccades (º/S)	0.5827	0.4627	0.3789
Standard Deviation of Velocity of Microsaccades	0.7233	0.4517	0.801
Peak Velocity-Microsaccades (º/S)	0.4322	0.2266	0.2834
Standard Deviation of Peak Velocity-Microsaccades	0.4867	0.4774	0.1110
Drift Amplitude (º)	0.4411	0.9431	0.8393
Standard Deviation Of Drift Amplitude	0.7924	0.2952	0.7877
Drift Velocity (º/S)	0.1874	0.1634	0.6870
Standard Deviation Of Drift Velocity	0.4826	0.1846	0.9965
Peak Of Velocity-Drift (º/S)	0.5126	0.3143	0.9752
Standard Deviation Of Peak Of Velocity-Drift	0.3449	0.0964	0.2402
Square Wave Jerks - Amplitude (º)	0.2016	0.5075	0.1202
Standard Deviation Of Square Wave Jerks - Amplitude	0.2428	0.1495	0.2019
Square Wave Jerks -Time (Ms)	0.6696	0.4187	0.3708
Standard Deviation Of Square Wave Jerks-Time	0.8161	0.9955	0.4478

P values for interaction between sex distribution and eye-movement parameters are shown, taking control group as reference. NMHE, MHE, patients without or with minimal hepatic encephalopathy, respectively; SD, Standard Deviation.
