# Supplementary material for: Eye movement analysis detects early neurological alterations in cirrhotic patients classified as not having minimal hepatic encephalopathy by the Psychometric Hepatic Encephalopathy Score
Source: PLoS One. 2026 Sep 15;21(9):e0358353. doi: 10.1371/journal.pone.0358353 (PMC13577538; doi:10.1371/journal.pone.0358353)
Supplement: Supplementary file 11 [file pone.0358353.s011.pdf]

Eye movement analysis detects early neurological alterations in cirrhotic patients classified as not having minimal hepatic encephalopathy by the Psychometric Hepatic Encephalopathy Score

Juan-José Gallego¶, Franc Casanova-Ferrer¶, Alessandra Fiorillo, Adrià López-Gramaje, Danna Campos, Amparo Urios, Javier Megías, Teresa San-Miguel, Montserrat Alcahuz-Griñán, Lucía Durbán, Elena Kosenko, Cecilia E. García-Cena, Carmina Montoliu*

¶These authors contributed equally to this work. *Corresponding author: Carmina Montoliu

S11 Table. Analysis of interaction between severity of liver damage and eye-movement parameters: visual-guided test.

	**Visual-guided test (horizontal)**				**Visual-guided test (vertical)**			
**Variable**	**Child Pugh** x **Early-MHE**	**Child Pugh** x **MHE**	**MELD x Early-MHE**	**MELD x MHE**	**Child Pugh** x **Early-MHE**	**Child Pugh** x **MHE**	**MELD x Early-MHE**	**MELD x MHE**
Latency (ms)	0.4345	0.4285	0.2115	0.6748	0.0956	0.6305	0.3328	0.7853
Standard Deviation of Latency	0.8352	0.5083	0.2099	0.4663	0.0508	0.2769	0.1125	0.4124
Gain	0.5805	0.5969	0.9445	0.9398	0.368	0.5134	0.931	0.8604
Standard Deviation of Gain	0.3987	0.4285	0.173	0.6202	0.7725	0.6845	0.2689	0.5064
Peak of velocity (º/ms)	0.0858	0.1847	0.8144	0.6807	0.0806	0.1028	0.2074	0.3555
Standard Deviation of Peak of Velocity	0.0587	**0.0489**	0.6414	0.3523	0.3539	0.7477	0.4215	0.8636
Positive Error	0.4553	0.3758	0.5027	0.5427	0.6641	0.5553	0.2613	0.6082
Standard Deviation of Positive Error	0.9933	0.5062	0.6927	0.2252	0.9498	0.3182	0.6562	0.4613
Negative Error	0.1759	0.2865	0.7436	0.2540	0.3718	0.5423	0.0865	0.5119
Standard Deviation of Negative Error	0.7023	0.9348	0.7981	0.4031	0.532	0.6731	0.7562	0.9929
Blinks	0.8066	0.876	0.5724	0.8191	0.231	0.9643	0.6852	0.7257
Anticipated Saccades	0.7397	**0.0405**	0.1526	**0.0437**	0.1918	0.0527	0.2955	0.1789
Return latency (ms)	0.7303	**0.0498**	0.4551	0.0732	0.1137	0.5655	0.9256	0.6489
Standard Deviation of Return Latency	0.684	0.0920	0.2724	0.1274	0.1987	0.325	0.4667	0.7221
Return Peak of Velocity (º/ms)	**0.0034**	**0.0329**	0.4382	0.6201	0.1118	0.2183	0.0899	0.2926
Standard Deviation of Return Peak of Velocity	**0.0145**	0.0991	0.4845	0.7877	0.3874	0.3912	0.9075	0.7149

Significant interactions are in bold. MELD, Model End-stage Liver Disease. NMHE, MHE, patients without or with minimal hepatic encephalopathy, respectively.
