# Supplementary material for: Eye movement analysis detects early neurological alterations in cirrhotic patients classified as not having minimal hepatic encephalopathy by the Psychometric Hepatic Encephalopathy Score
Source: PLoS One. 2026 Sep 15;21(9):e0358353. doi: 10.1371/journal.pone.0358353 (PMC13577538; doi:10.1371/journal.pone.0358353)
Supplement: Supplementary file 12 [file pone.0358353.s012.pdf]

Eye movement analysis detects early neurological alterations in cirrhotic patients classified as not having minimal hepatic encephalopathy by the Psychometric Hepatic Encephalopathy Score

Juan-José Gallego¶, Franc Casanova-Ferrer¶, Alessandra Fiorillo, Adrià López-Gramaje, Danna Campos, Amparo Urios, Javier Megías, Teresa San-Miguel, Montserrat Alcahuz-Griñán, Lucía Durbán, Elena Kosenko, Cecilia E. García-Cena, Carmina Montoliu*

¶These authors contributed equally to this work. *Corresponding author: Carmina Montoliu

S12 Table. Analysis of interaction between severity of liver damage and eye-movement parameters: memory-guided test.

Variable	Memory-guided test (horizontal)				Memory-guided test (vertical)			
	**Child Pugh x Early-MHE**	**Child Pugh x MHE**	**MELD x Early-MHE**	**MELD x MHE**	**Child Pugh x Early-MHE**	**Child Pugh x MHE**	**MELD x Early-MHE**	**MELD x MHE**
Latency (ms)	0.0965	0.0876	0.8391	0.5454	0.7238	0.9713	0.5153	0.2000
Standard Deviation of Latency	0.0835	0.0848	0.6975	0.5964	0.7171	0.0818	0.3011	0.5003
Gain	0.3752	0.608	0.2213	0.2570	0.2370	0.8096	0.8144	0.7847
Standard Deviation of Gain	0.8143	0.9913	0.3036	0.9793	0.6377	0.2926	0.9556	0.4727
Peak of velocity (º/ms)	0.8040	0.2056	0.723	0.6738	**0.0394**	**0.0093**	0.1976	**0.0497**
Standard Deviation of Peak of Velocity	0.9556	**0.0238**	0.8256	0.3844	0.0447	**0.0470**	0.0413	**0.0034**
Positive Errors	0.3521	0.7413	0.1248	0.3789	0.8183	0.1378	0.0813	0.9837
Standard Deviation of Positive Errors	**0.0347**	0.6953	**0.0098**	0.4212	0.6437	0.4186	0.6170	**0.0435**
Negative Errors	0.6335	0.9535	0.3057	0.4054	0.6929	0.1893	0.3918	0.2208
Standard Deviation of Negative Errors	0.4032	0.1876	0.9854	0.5364	0.9517	**0.0004**	0.5995	**0.0143**
Blinks	0.2096	0.9808	0.8903	0.5757	0.2267	0.7305	0.6578	0.7280
Anticipated Saccades	0.9049	0.8194	0.3776	0.1288	0.7746	0.8958	0.8502	0.7230
Correct saccades	0.1588	**0.0196**	0.5455	**0.0294**	0.3584	0.1750	0.6334	0.6143
Return latency (ms)	0.2201	0.1062	0.9214	0.7981	0.5918	0.6229	0.6994	0.8121
Standard Deviation of Return Latency	0.9333	0.6816	0.3331	0.9868	0.8360	0.7938	0.3596	0.3280
Return Peak of Velocity (º/ms)	0.7314	0.3895	0.9362	0.1957	0.1904	**0.0274**	**0.0166**	**0.0228**
Standard Deviation of Return Peak of Velocity	0.8924	0.5989	0.8071	0.9004	0.8876	0.6129	0.3967	0.2914

Significant interactions are in bold. MELD, Model End-stage Liver Disease. NMHE, MHE, patients without or with minimal hepatic encephalopathy, respectively.
