# Supplementary material for: Eye movement analysis detects early neurological alterations in cirrhotic patients classified as not having minimal hepatic encephalopathy by the Psychometric Hepatic Encephalopathy Score
Source: PLoS One. 2026 Sep 15;21(9):e0358353. doi: 10.1371/journal.pone.0358353 (PMC13577538; doi:10.1371/journal.pone.0358353)
Supplement: Supplementary file 13 [file pone.0358353.s013.pdf]

Eye movement analysis detects early neurological alterations in cirrhotic patients classified as not having minimal hepatic encephalopathy by the Psychometric Hepatic Encephalopathy Score

Juan-José Gallego¶, Franc Casanova-Ferrer¶, Alessandra Fiorillo, Adrià López-Gramaje, Danna Campos, Amparo Urios, Javier Megías, Teresa San-Miguel, Montserrat Alcahuz-Griñán, Lucía Durbán, Elena Kosenko, Cecilia E. García-Cena, Carmina Montoliu*

¶These authors contributed equally to this work. *Corresponding author: Carmina Montoliu

S13 Table. Analysis of interaction between severity of liver damage and eye-movement parameters: Smooth Pursuit tests.

	**Smooth pursuit test (Horizontal)**				**Smooth pursuit test (vertical)**				**Smooth pursuit test (Sinusoidal)**			
**Variable**	**Child Pugh x Early-MHE**	**Child Pugh x MHE**	**MELD x Early-MHE**	**MELD x MHE**	**Child Pugh x Early-MHE**	**Child Pugh x MHE**	**MELD x Early-MHE**	**MELD x MHE**	**Child Pugh x Early-MHE**	**Child Pugh x MHE**	**MELD x Early-MHE**	**MELD x MHE**
Blinks	0.5827	0.6964	0.4281	0.6457	0.3097	0.9230	0.5599	0.6779	0.1763	0.6905	0.6577	0.7978
Catch-up saccades	**0.0057**	**0.0334**	**0.0163**	**0.0053**	**0.0044**	**0.0050**	0.3559	0.0727	**0.0007**	0.1215	**0.0010**	**0.0373**
Back-up saccades	0.6080	0.4383	0.2180	0.7789	0.6581	0.6658	0.9052	0.1889	0.5286	0.6335	0.3734	0.9799
Square Wave Jerks	0.3501	0.1240	0.2696	0.1391	0.8056	0.9127	0.4477	0.9370	0.2400	0.5869	0.3802	0.4075
Pursuit Time (%)	**0.0001**	**0.0001**	0.0538	0.1138	**0.0002**	**0.0001**	0.1362	0.0713	0.0791	0.5322	0.9264	0.9922
Latency (ms)	0.1384	**0.0124**	0.6039	0.3447	0.8357	0.0977	0.4178	0.9427	0.6802	0.9664	0.4657	0.7724
Total Mean Squared Error of Position	**0.0014**	**0.0001**	0.9462	0.3859	0.8669	0.6933	0.8829	0.6022	0.2443	0.7172	0.1726	0.2991
Pursuit Mean Squared Error of Position	**0.0011**	**0.0001**	0.8827	0.3523	0.7906	0.6768	0.9309	0.6204	0.2505	0.7138	0.1821	0.3225
Gain	0.2227	**0.0384**	0.1350	0.8055	**0.0001**	**0.0001**	0.0633	**0.0371**	0.7529	0.6631	0.5156	0.1057
Pursuit Mean Squared Error of Velocity	0.5764	0.4689	0.2603	0.4619	**0.0001**	**0.0001**	0.1984	**0.0268**	0.4106	0.5836	0.3403	0.7136

Significant interactions are in bold. MELD, Model End-stage Liver Disease. NMHE, MHE, patients without or with minimal hepatic encephalopathy, respectively.
