# Supplementary material for: Eye movement analysis detects early neurological alterations in cirrhotic patients classified as not having minimal hepatic encephalopathy by the Psychometric Hepatic Encephalopathy Score
Source: PLoS One. 2026 Sep 15;21(9):e0358353. doi: 10.1371/journal.pone.0358353 (PMC13577538; doi:10.1371/journal.pone.0358353)
Supplement: Supplementary file 14 [file pone.0358353.s014.pdf]

Eye movement analysis detects early neurological alterations in cirrhotic patients classified as not having minimal hepatic encephalopathy by the Psychometric Hepatic Encephalopathy Score

Juan-José Gallego¶, Franc Casanova-Ferrer¶, Alessandra Fiorillo, Adrià López-Gramaje, Danna Campos, Amparo Urios, Javier Megías, Teresa San-Miguel, Montserrat Alcahuz-Griñán, Lucía Durbán, Elena Kosenko, Cecilia E. García-Cena, Carmina Montoliu*

¶These authors contributed equally to this work. *Corresponding author: Carmina Montoliu

S14 Table. Analysis of interaction between severity of liver damage and eye-movement parameters: Antisaccades test.

Variable	Antisaccades test (horizontal)				Antisaccades test (vertical)			
	Child Pugh x Early-MHE	Child Pugh x MHE	MELD x Early-MHE	MELD x MHE	Child Pugh x Early-MHE	Child Pugh x MHE	MELD x Early-MHE	MELD x MHE
Latency (ms)	0.7045	0.0807	0.3361	0.7849	0.9950	0.7963	0.9415	0.4156
Standard Deviation of Latency	0.1701	0.8200	0.1964	0.7991	0.9574	0.8675	0.9167	0.3809
Latency of reflexive saccades (ms)	0.9331	0.7199	0.3848	0.5592	0.7816	0.1348	0.7905	0.3550
SD of Latency of reflexive saccades	0.4519	0.6391	0.8564	0.3408	0.4986	0.1093	0.6814	0.6704
Duration of reflexive saccades (ms)	0.5775	0.7212	0.891	0.9195	0.8092	0.1452	0.7270	0.1399
SD of Duration of reflexive saccades	0.5174	0.8115	0.8716	0.5421	0.5564	0.9874	0.8624	0.1970
Positive Errors	0.6590	0.8669	0.9215	0.8256	0.8545	0.3734	0.4911	**0.0119**
SD of Positive Errors	0.5699	0.8419	0.9278	0.4281	0.8485	0.2521	0.9109	**0.0066**
Negative Errors	0.1980	0.8013	0.1590	0.0769	0.6416	0.1524	0.8684	0.2624
SD of Negative Errors	0.2261	0.5586	0.1086	0.1029	0.1201	0.2196	0.4748	0.8754
Blinks	0.4182	0.1741	0.4355	0.5233	0.2248	0.9942	0.7947	0.4315
Number of correct antisaccades	0.0701	0.0554	0.4855	0.8269	**0.0315**	**0.0148**	0.7202	0.3924
Number of corrected antisaccades	0.0637	0.1873	0.6554	0.9407	**0.0132**	0.1049	0.3195	0.9074
Number of incorrect antisaccades	0.5262	0.8178	0.3183	0.4632	0.6120	0.5978	0.0997	0.3794
Number of second order reflexive saccades	0.1156	0.9282	0.3158	0.9424	0.3713	0.5376	0.8539	0.9806
Number of Anticipated Saccades	0.5658	0.9873	0.6487	0.1907	0.8464	0.9950	0.2500	0.6571
Return latency (ms)	0.9122	0.9195	0.4575	0.2388	0.6080	0.2317	0.8558	**0.0191**
SD of Return Latency	0.8960	0.8686	0.9587	0.3194	0.2599	0.6728	0.6890	**0.0396**
Return Peak of velocity (°/ms)	0.7939	0.5163	0.4817	0.4141	0.8990	0.5927	0.0982	**0.0030**
SD of Return Peak of Velocity	0.2164	0.3402	0.1238	0.3475	0.7695	0.7762	0.1453	0.0999

Significant interactions are in bold. MELD, Model End-stage Liver Disease. NMHE, MHE, patients without or with minimal hepatic encephalopathy, respectively. SD, Standard Deviation.
