# Supplementary material for: Eye movement analysis detects early neurological alterations in cirrhotic patients classified as not having minimal hepatic encephalopathy by the Psychometric Hepatic Encephalopathy Score
Source: PLoS One. 2026 Sep 15;21(9):e0358353. doi: 10.1371/journal.pone.0358353 (PMC13577538; doi:10.1371/journal.pone.0358353)
Supplement: Supplementary file 15 [file pone.0358353.s015.pdf]

Eye movement analysis detects early neurological alterations in cirrhotic patients classified as not having minimal hepatic encephalopathy by the Psychometric Hepatic Encephalopathy Score

Juan-José Gallego¶, Franc Casanova-Ferrer¶, Alessandra Fiorillo, Adrià López-Gramaje, Danna Campos, Amparo Urios, Javier Megías, Teresa San-Miguel, Montserrat Alcahuz-Griñán, Lucía Durbán, Elena Kosenko, Cecilia E. García-Cena, Carmina Montoliu*

¶These authors contributed equally to this work. *Corresponding author: Carmina Montoliu

S15 Table. Analysis of interaction between severity of liver damage and eye-movement parameters: Fixation test.

Variable	Child Pugh x Early-MHE	Child Pugh x MHE	MELD x Early-MHE	MELD x MHE
Blinks	0.5816	0.8250	0.7459	0.3822
Saccades	0.1226	0.3390	**0.0361**	0.7698
Microsaccades	0.1437	0.2595	**0.0460**	0.0913
Number of Drifts	0.2732	0.2949	0.0777	**0.0471**
Monophasic- Square Wave Jerks	0.1745	0.3033	0.0511	0.5108
Biphasic- Square Wave Jerks	0.3518	0.3008	0.1100	0.2522
Distractions	0.9045	0.3630	0.8867	0.3541
BCEA (º)	0.4247	0.8914	0.6798	0.9579
Ox (Horizontal Standard Deviation) (º)	0.5846	0.6345	0.5052	0.5481
Oy (Vertical Standard Deviation) (º)	0.9224	0.3103	0.9062	0.5037
Absolute Centroid X (Horizontal) (º)	0.6049	0.4141	0.5869	0.3832
Absolute Centroid Y (Vertical) (º)	0.5680	0.8697	0.3685	0.7725
Microsaccades Amplitude (º)	0.7988	0.9713	**0.0498**	0.0855
Standard Deviation of Microsaccades Amplitude	0.5784	0.9390	0.2995	0.9588
Velocity of Microsaccades (º/S)	0.7066	0.6803	0.4139	0.3703
Standard Deviation of Velocity of Microsaccades	0.4321	0.7941	0.3923	0.7177
Peak Velocity-Microsaccades (º/S)	0.9547	0.8376	0.2551	0.1680
Standard Deviation of Peak Velocity-Microsaccades	0.4458	0.5389	0.1947	0.0782
Drift Amplitude (º)	0.1365	0.2276	0.7669	0.2213
Standard Deviation of Drift Amplitude	0.2209	0.1844	0.2157	0.9449
Drift Velocity (º/S)	0.4377	0.4991	**0.0270**	0.3258
Standard Deviation of Drift Velocity	0.0652	0.1013	0.1364	0.8477
Peak of Velocity-Drift (º/S)	0.9931	0.9703	0.5215	0.3019
Standard Deviation of Peak Of Velocity-Drift	0.8735	0.5552	0.0413	0.3412
Square Wave Jerks - Amplitude (º)	0.5105	0.9238	0.7360	0.9735
Standard Deviation of Square Wave Jerks - Amplitude	0.2165	0.4906	0.2362	0.9935
Square Wave Jerks -Time (ms)	**0.0408**	0.0565	0.8343	0.7949
Standard Deviation of Square Wave Jerks-Time	0.7445	0.3734	0.6070	0.8871

Significant interactions are in bold. MELD, Model End-stage Liver Disease. NMHE, MHE, patients without or with minimal hepatic encephalopathy, respectively
