# Supplementary material for: Eye movement analysis detects early neurological alterations in cirrhotic patients classified as not having minimal hepatic encephalopathy by the Psychometric Hepatic Encephalopathy Score
Source: PLoS One. 2026 Sep 15;21(9):e0358353. doi: 10.1371/journal.pone.0358353 (PMC13577538; doi:10.1371/journal.pone.0358353)
Supplement: Supplementary file 16 [file pone.0358353.s016.pdf]

Eye movement analysis detects early neurological alterations in cirrhotic patients classified as not having minimal hepatic encephalopathy by the Psychometric Hepatic Encephalopathy Score

Juan-José Gallego¶, Franc Casanova-Ferrer¶, Alessandra Fiorillo, Adrià López-Gramaje, Danna Campos, Amparo Urios, Javier Megías, Teresa San-Miguel, Montserrat Alcahuz-Griñán, Lucía Durbán, Elena Kosenko, Cecilia E. García-Cena, Carmina Montoliu*

¶These authors contributed equally to this work. *Corresponding author: Carmina Montoliu

S16 Table. Diagnostic accuracy of eye movement variables to detect mild cognitive alterations in cirrhotic patients by univariate logistic regression analyses.

Variable	Test	OR (95% CI)	p value
Latency of reflexive saccades	HA	1.006 (1.003 – 1.009)	p < 0.001
Drift amplitude	F	2439 (3.65 - 1629239)	0.02
Number of incorrect antisaccades	HA	1.286 (1.061 – 1.559)	0.01
Number of anticipated antisaccades	HA	0.774 (0.630 – 0.880)	0.001
SD of negative error	HA	1.304 (1.068 – 1.592)	0.009
Duration of reflexive saccades	HA	1.007 (1.003 – 1.010)	0.001
BCEA	F	1.329 (0.996 – 1.773)	0.054
Latency of reflexive saccades	VA	1.005 (1.002 – 1.008)	0.002
SD of latency of reflexive saccades	HA	1.008 (1.004 – 1.014)	0.003
Ox (horizontal standard deviation)	F	2.361 (0.994 – 5.907)	0.066
SD of duration of reflexive saccades	HA	1.008 (1.003 – 1.013)	0.004
Positive error	VA	1.705 (1.160 – 2.505)	0.007
Number of correct saccades	VMS	0.766 (0.652 – 0.900)	0.001
Number of correct saccades	HMS	0.853 (0.765 – 0.952)	0.005
Number of incorrect antisaccades	VA	1.388 (1.047 – 1.841)	0.023
Number of anticipated antisaccades	VA	0.686 (0.540 – 0.871)	0.002
Latency	VA	1.067 (1.001 – 1.010)	0.013
Number of corrected antisaccades	HA	1.287 (0.993 – 1.667)	0.056
Positive error	HA	1.204 (1.032 – 1.406)	0.02
SD of latency	HA	1.010 (1.01 – 1.018)	0.02
Pursuit mean squared error of velocity	SSP	1.175 (0.994 – 1.389)	0.058
Square wave jerks—amplitude	F	3.687 (0.777 – 19.25)	0.099
Saccades	F	1.031 (0.981 – 1.081)	0.238
SD of return latency	HA	1.008 (1.002 – 1.014)	0.007
Total mean squared error of position	VSP	1.397 (1.008 – 1.937)	0.045
Pursuit mean squared error of position	VSP	1.406 (1.001 – 1.975)	0.049
Oy (vertical standard deviation)	F	0.950 (0.778 – 1.159)	0.612
Negative error	HA	0.894 (0.800 – 0.998)	0.046
SD of positive error	HA	1.246 (0.987 – 1.574)	0.064
Latency	VS	1.012 (1.000 – 1.025)	0.042
Number of corrected antisaccades	VA	1.714 (1.138 – 2.581)	0.010
Gain	HSP	0.093 (0.007 – 1.216)	0.07
Cath-up saccades	SSP	1.058 (0.998 – 1.121)	0.057
Duration of reflexive saccades	VA	1.004 (1.000 – 1.008)	0.031
Pursuit mean squared error of position	SSP	1.130 (0.912 – 1.400)	0.264
Total mean squared error of position	SSP	1.131 (0.972 – 1.395)	0.250
Number of correct antisaccades	HA	0.879 (0.780 – 0.989)	0.032
Square wave jerks	SSP	1.118 (0.987 – 1.266)	0.080
Negative error	VA	0.7956 (0.661 – 0.954)	0.014
Microsaccades amplitude	F	13.34 (0.660 – 269.4)	0.091
Return peak of velocity	VA	1.004 (0.999 – 1.09)	0.089
Biphasic- square wave jerks	F	1.158 (0.958 – 1.398)	0.129
Number of anticipated saccades	VS	0.557 (0.361 – 0.859)	0.008

SD, standard deviation; HA, horizontal antisaccades; F, fixation; VA, vertical antisaccades; VMS, vertical memory-guided saccades; HMS; horizontal memory-guided saccades; SSP, sinusoidal smooth pursuit; VSP, vertical smooth pursuit; VS, vertical saccades; HSP, horizontal smooth pursuit.
