# Supplementary material for: Eye movement analysis detects early neurological alterations in cirrhotic patients classified as not having minimal hepatic encephalopathy by the Psychometric Hepatic Encephalopathy Score
Source: PLoS One. 2026 Sep 15;21(9):e0358353. doi: 10.1371/journal.pone.0358353 (PMC13577538; doi:10.1371/journal.pone.0358353)
Supplement: Supplementary file 17 [file pone.0358353.s017.pdf]

Eye movement analysis detects early neurological alterations in cirrhotic patients classified as not having minimal hepatic encephalopathy by the Psychometric Hepatic Encephalopathy Score

Juan-José Gallego¶, Franc Casanova-Ferrer¶, Alessandra Fiorillo, Adrià López-Gramaje, Danna Campos, Amparo Urios, Javier Megías, Teresa San-Miguel, Montserrat Alcahuz-Griñán, Lucía Durbán, Elena Kosenko, Cecilia E. García-Cena, Carmina Montoliu*

¶These authors contributed equally to this work. *Corresponding author: Carmina Montoliu

S17 Table. Diagnostic accuracy of eye movement variables to detect mild cognitive alterations in cirrhotic patients by ROC analyses.

Variable	Test	AUROC (95% CI)	p value	Cutoff	Sens.	Spec.
Latency of reflexive saccades	HA	0.772 (0.678 - 0.866)	p < 0.001	676.75[a]	54.84	88.24
Drift amplitude	F	0.747 (0.602 - 0.892)	0.002	0.16[b]	88.10	57.89
Number of incorrect antisaccades	HA	0.734 (0.641 - 0.826)	p < 0.001	1.00	67.14	77.78
Number of anticipated antisaccades	HA	0.734 (0.635 - 0.833)	p < 0.001	1.00	65.71	69.44
SD of negative error	HA	0.734 (0.613 - 0.855)	0.001	2.55	75,00	80,00
Duration of reflexive saccades	HA	0.730 (0.628 - 0.832)	p < 0.001	276.09[a]	63.93	73.53
BCEA	F	0.722 (0.617 - 0.828)	p < 0.001	0.68[b]	66.18	72.73
Latency of reflexive saccades	VA	0.721 (0.613 - 0.829)	0.001	818.86[a]	35.19	100.00
SD of latency of reflexive saccades	HA	0.720 (0.610 - 0.830)	0.001	165.89	66.67	71.88
Ox (horizontal standard deviation)	F	0.718 (0.609 - 0.827)	p < 0.001	0.24[b]	79.41	57.58
SD of duration of reflexive saccades	HA	0.710 (0.594 - 0.826)	0.001	123.03	72.55	68.75
Positive error	VA	0.704 (0.583 - 0.826)	0.004	3.11	42.86	89.66
Number of correct saccades	VMS	0.698 (0.595 - 0.801)	0.001	6.00	66.67	72.97
Number of correct saccades	HMS	0.689 (0.580 - 0.797)	0.001	9.00	63.24	71.05
Number of incorrect antisaccades	VA	0.681 (0.587 - 0.776)	0.001	1.00	60.29	72.97
Number of anticipated antisaccades	VA	0.680 (0.571 - 0.789)	0.002	1.00	70.59	62.16
Latency	VA	0.674 (0.542 - 0.806)	0.019	539.97[a]	37.84	92.59
Number of corrected antisaccades	HA	0.673 (0.570 - 0.775)	0.002	1.00	65.71	66.67
Positive error	HA	0.671 (0.552 - 0.789)	0.008	2.43	74.07	60.61
SD of latency	HA	0.671 (0.511 - 0.831)	0.043	81.17	69.57	64,00
Pursuit mean squared error of velocity	SSP	0.663 (0.541 - 0.785)	0.011	8.96	59.32	70.97
Square wave jerks—amplitude	F	0.663 (0.512 - 0.815)	0.048	0.55[b]	82.98	52.94
Saccades	F	0.662 (0.544 - 0.781)	0.007	1.5	72.06	58.82
SD of return latency	HA	0.653 (0.546 - 0.760)	0.011	105.51	69.23	61.11
Total mean squared error of position	VSP	0.651 (0.532 - 0.770)	0.016	1.07	91.18	38.71
Pursuit mean squared error of position	VSP	0.651 (0.531 - 0.772)	0.016	1.03	91.18	38.71
Oy (vertical standard deviation)	F	0.648 (0.535 - 0.760)	0.017	0.3[b]	63.24	69.7
Negative error	HA	0.648 (0.529 - 0.767)	0.022	-3.93	60.00	78.13
SD of positive error	HA	0.648 (0.521 - 0.774)	0.029	1.9	72.09	57.58
Latency	VS	0.646 (0.529 - 0.764)	0.019	236.05[a]	87.5	39.39
Number of corrected antisaccades	VA	0.643 (0.548 - 0.737)	0.009	2.00	38.24	91.89
Gain	HSP	0.643 (0.520 - 0.767)	0.019	0.89	78.87	57.58
Cath-up saccades	SSP	0.637 (0.509 - 0.765)	0.032	14.00	83.05	43.75
Duration of reflexive saccades	VA	0.636 (0.517 - 0.755)	0.036	231.45[a]	55.56	71.88
Pursuit mean squared error of position	SSP	0.635 (0.508 - 0.761)	0.037	1.48	86.44	41.94
Total mean squared error of position	SSP	0.632 (0.505 - 0.759)	0.041	2.24	72.88	54.84
Number of correct antisaccades	HA	0.629 (0.517 - 0.740)	0.030	4.00	51.43	72.22
Square wave jerks	SSP	0.628 (0.503 - 0.752)	0.043	1.00	93.22	34.38
Negative error	VA	0.626 (0.501 - 0.741)	0.049	-6.35	30.91	100.00
Microsaccades amplitude	F	0.625 (0.498 - 0.752)	0.048	0.37[b]	72.31	58.06
Return peak of velocity	VA	0.623 (0.506 - 0.741)	0.042	193.38[c]	68.25	58.33
Biphasic- square wave jerks	F	0.612 (0.511 - 0.712)	0.041	1.00	50.00	73.53
Number of anticipated saccades	VS	0.609 (0.284 - 0.499)	0.036	1.00	90.91	31.43

Receiver operating characteristic (ROC) curves for sensitivity and specificity to detect early cognitive alterations in the cohort patients using eye movement parameters. [a]: ms; [b]: º; [c]: º/ms. AUROC, area under the receiver operating curve; CI, confidence interval; Sens: sensitivity; Spec: specificity; BCEA, bivariate contour ellipse area; SD, standard deviation; HA, horizontal antisaccades; F, fixation; VA, vertical antisaccades; VMS, vertical memory-guided saccades; HMS; horizontal memory-guided saccades; SSP, sinusoidal smooth pursuit; VSP, vertical smooth pursuit; VS, vertical saccades; HSP, horizontal smooth pursuit.
