# Supplementary material for: Eye movement analysis detects early neurological alterations in cirrhotic patients classified as not having minimal hepatic encephalopathy by the Psychometric Hepatic Encephalopathy Score
Source: PLoS One. 2026 Sep 15;21(9):e0358353. doi: 10.1371/journal.pone.0358353 (PMC13577538; doi:10.1371/journal.pone.0358353)
Supplement: Supplementary file 18 [file pone.0358353.s018.pdf]

Eye movement analysis detects early neurological alterations in cirrhotic patients classified as not having minimal hepatic encephalopathy by the Psychometric Hepatic Encephalopathy Score

Juan-José Gallego¶, Franc Casanova-Ferrer¶, Alessandra Fiorillo, Adrià López-Gramaje, Danna Campos, Amparo Urios, Javier Megías, Teresa San-Miguel, Montserrat Alcahuz-Griñán, Lucía Durbán, Elena Kosenko, Cecilia E. García-Cena, Carmina Montoliu*

¶These authors contributed equally to this work. *Corresponding author: Carmina Montoliu

S18 Table. Comparison of antisaccade parameters included in the model between patients with alcoholic etiology and with other etiologies, in the groups of patients without MHE and Early-MHE

	NMHE patients			Early-MHE patients		
**Variable**	**Alcohol**	**Other etiologies**	***P* alcohol *vs.* other etiologies**	**Alcohol**	**Other etiologies**	***P* alcohol *vs.* other etiologies**
Latency of reflexive saccades (ms)	554 (41)	595 (43)	0.523	750 (51)**	766 (73)$^{\alpha}$	0.853
SD of latency of reflexive saccades	138 (22)	132 (16)	0.820	196 (29)	271 (65)$^{\alpha}$	0.243
Duration of reflexive saccades (ms)	221 (24)	243 (27)	0.582	352 (43)*	341 (47)	0.867
SD of duration of reflexive saccades	123 (24)	119 (18)	0.898	179 (28)	225 (64)$^{\alpha}$	0.460
Number of incorrect antisaccades	0.20 (0.14)	0.78 (0.35)	0.293	4.2 (0.9)***	1.12 (0.35)	0.020
Number of anticipated antisaccades	1.13 (0.65)	1.13 (0.38)	0.690	1.30 (0.34)	1.54 (0.49)	0.690

Data are expressed as mean (SEM). Values significantly different from NMHE patients with alcohol etiology are indicated by asterisks (*). Values significantly different from NMHE patients with other etiologies are indicated by ($^{\alpha}$) */$^{\alpha}$ p<0.05; ** p<0.01; ***p<0.001.
