# Supplementary material for: Eye movement analysis detects early neurological alterations in cirrhotic patients classified as not having minimal hepatic encephalopathy by the Psychometric Hepatic Encephalopathy Score
Source: PLoS One. 2026 Sep 15;21(9):e0358353. doi: 10.1371/journal.pone.0358353 (PMC13577538; doi:10.1371/journal.pone.0358353)
Supplement: Supplementary file 19 [file pone.0358353.s019.pdf]

Eye movement analysis detects early neurological alterations in cirrhotic patients classified as not having minimal hepatic encephalopathy by the Psychometric Hepatic Encephalopathy Score

Juan-José Gallego¶, Franc Casanova-Ferrer¶, Alessandra Fiorillo, Adrià López-Gramaje, Danna Campos, Amparo Urios, Javier Megías, Teresa San-Miguel, Montserrat Alcahuz-Griñán, Lucía Durbán, Elena Kosenko, Cecilia E. García-Cena, Carmina Montoliu*

¶These authors contributed equally to this work. *Corresponding author: Carmina Montoliu

S19 Table. Comparison of antisaccade parameters included in the model between patients with and without diabetes mellitus in the groups of patients without MHE and Early-MHE.

	NMHE patients			Early-MHE patients		
**Variable**	**NO-DM**	**DM**	***P* DM *vs.* NO-DM**	**NO-DM**	**DM**	***P* DM *vs.* NO-DM**
Latency of reflexive saccades (ms)	594 (33)	452 (63)	0.160	730 (48)*	735 (49)$^{\alpha\alpha}$	0.947
SD of latency of reflexive saccades	135 (14)	125 (31)	0.808	263 (41)**	183 (33)	0.199
Duration of reflexive saccades (ms)	242 (20)	164 (37)	0.206	342 (39)*	346 (39)$^{\alpha}$	0.956
SD of duration of reflexive saccades	124 (15)	99 (43)	0.589	225 (40)*	165 (24)	0.310
Number of incorrect antisaccades	0.62 (0.24)	0.0 (0.0)	0.560	3.74 (0.81)***	1.17 (0.39)	0.105
Number of anticipated antisaccades	1.26 (0.37)	0.0 (0.0)	0.259	1.93 (0.42)	1.0 (0.3)	0.172

Data are expressed as mean (SEM). NMHE, patients without m inimal hepatic encephalopathy, NO-DM: Without diabetes; DM: With diabetes mellitus. Values significantly different from NMHE patients without diabetes are indicated by (*). Values significantly different from NMHE patients with diabetes are indicated by ($^{\alpha}$). */ $^{\alpha}$ $p<0.05$; **/$^{\alpha\alpha}$ $p<0.01$; ***$p<0.001$
